# Apolipoprotein M expression modifies the sphingolipid landscape in murine blood and lymph

**DOI:** 10.3389/fimmu.2025.1572959

**Published:** 2025-05-02

**Authors:** Victoria A. Blaho, Joshua T. Minyard

**Affiliations:** Cancer Metabolism and Microenvironment Program, NCI-designated Cancer Center, Sanford Burnham Prebys Medical Discovery Institute, La Jolla, CA, United States

**Keywords:** sphingolipid, lipidomics, lymph, blood, apolipoprotein M, sphingosine 1-phosphate, high-density lipoprotein (HDL), inflammation

## Abstract

Members of the diverse family of sphingolipids (SPL), such as ceramides (Cer) and sphingomyelins (SM), are well-known structural and bioactive signaling molecules. A key SPL family member and critical signaling lipid, sphingosine 1-phosphate (S1P), is carried in blood primarily by its “chaperone” protein apolipoprotein M (ApoM) on high-density lipoprotein (HDL) particles. S1P has been shown to regulate diverse biological pathways through specific G protein-coupled receptor signaling (GPCR) that can be modulated based upon chaperone: ApoM or albumin. Blood concentrations of ApoM itself are altered in human diseases such as coronary artery disease, type I and II diabetes, and systemic lupus erythematosus, diseases that have also been linked to changes in other SPL species; however, studies measuring molecules only in blood while neglecting lymph concentrations may be excluding clues to the physiology affected by multiorgan metabolic pathways. Comparing SM, dihydroSM, Cer, dihydroCer, α-hydroxy Cer (αOHCer), Cer 1-phosphate (C1P), sphingosine (Sph)/dihydroSph, S1P/dihydroS1P, and diacylglycerol (DAG) concentrations in wild-type mouse blood and lymph plasmas with those in mice lacking ApoM and mice expressing a human transgene of ApoM, we describe unanticipated differences between the blood and lymph sphingolipidomes and their ApoM-responsive lipid species. Of the 100 unique SPL species targeted, 97 were identified in blood and 94 in lymph. Some of the most striking findings were in lymph, where we identified αOHCer as a previously unidentified major SPL constituent. This report provides a unique resource and starting point for further investigations into the contributions of the circulating sphingolipidome to homeostasis and disease.

## Introduction

Sphingolipids (SPL) are a family of molecules with heterogeneous contributions to cellular structure and signaling. The SPL, sphingosine 1-phosphate (S1P), is a powerful signaling lipid formed by phosphorylation of sphingosine (Sph) derived from ceramide (Cer) and is carried in blood by two protein “chaperones”: apolipoprotein M (ApoM) and albumin (1, 2). At homeostasis, ApoM is found primarily on high-density lipoprotein (HDL) particles (1, 3). S1P regulates diverse biological pathways through activation of specific G protein-coupled receptors (GPCRs), S1P_1-5_ (4–6). In some instances, these signals may be modulated based upon chaperone: ApoM or albumin. For instance, ApoM- versus albumin-bound S1P has been shown to differentially regulate endothelial cell activation and subsequent vascular inflammation, as well as the suppression of lymphocyte progenitor proliferation versus other hematopoietic progenitor cell types (7, 8). Clinically, blood plasma or serum concentrations of both S1P and ApoM have been linked to human diseases such as sepsis, coronary artery disease (CAD), type I and II diabetes (T1/IID), systemic lupus erythematosus (SLE), and most recently, COVID (9–14).

Due to the difficulty of obtaining samples, studies of lymph fluid composition are comparatively rare versus those of blood, which is more readily obtained from animal and human subjects. Yet the lymphatic system is a critical component of the vascular system, transporting interstitial fluid, immune cells, and signaling molecules from the periphery to be reincorporated into the central venous system (15, 16). The lymphatics are also a major contributor to pharmacodynamics of molecules administered via subcutaneous, intranasal, and oral delivery routes and their subsequent blood pharmacokinetics (17–19). Measurements collected solely from blood while neglecting lymph concentrations may be excluding clues to physiology affected by multiorgan metabolic pathways. The role of S1P in lymphocyte egress is one such example. In 2002, it was shown that S1P receptor expression by lymphocytes was required for them to recognize high S1P concentrations in blood and lymph, drawing them out of lymph nodes and the spleen, which have tightly controlled, very low S1P concentrations (20, 21). Almost a decade later, HDL-bound ApoM was identified as a specific chaperone for approximately two-thirds of blood S1P versus the one-third carried by albumin (1, 22). Subsequently, mice lacking ApoM have blood plasma S1P concentrations one third to a quarter of wild-type (WT) samples (approximately 600-750 nM) (1, 7). Since previous studies repeatedly found that HDL- versus albumin-bound S1P had differential effects on signals transduced via S1P receptors, it was hypothesized that lymphocyte trafficking versus vascular permeability might similarly be modulated by S1P chaperone. However, loss of ApoM-bound S1P signaling did not affect lymphocyte egress *in vivo*, likely due in part to S1P concentrations in KO lymph that did not differ from WT (7).

**Figure 1:**
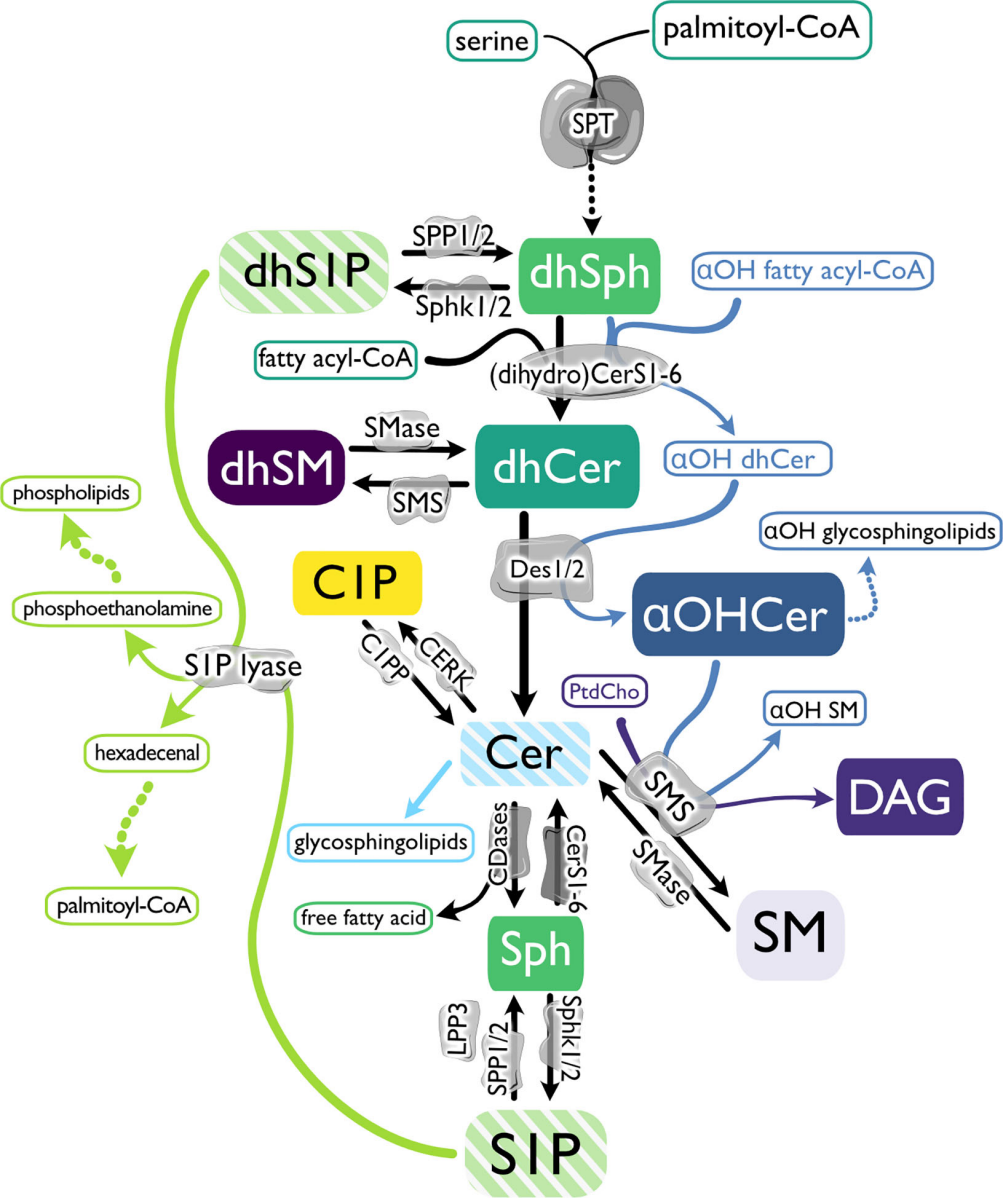
The biosynthetic pathway of sphingosine 1-phosphate and related molecules. *De novo* sphingolipid (SPL) metabolism begins with serine palmitoyltransferase (SPT)-mediated condensation of serine and palmitoyl-coenzyme A (CoA) and reduction of that product to generate dihydrosphingosine (dhSph). dhSph can then be phosphorylated to dihydrosphingosine 1-phosphate (dhS1P) by sphingosine kinases (Sphk1 and 2) or acylated by (dihydro) ceramide synthases ((dh)Cers) to form dihydroceramide (dhCer). If αOH-fatty acyl-CoAs are utilized by dhCers rather than non-hydroxylated fatty acyl-CoAs, α-hydroxylated Cer (αOHCer) are synthesized and can serve as the backbone for αOH glycosphingolipids. Dihydrosphingomyelin (dhSM) is synthesized from dhCer by sphingomyelin synthase (SMS), or desaturation of dhCer by dihydroceramide desaturases (Des 1 and 2) generates ceramide (Cer). Cer can then be phosphorylated by ceramide kinase (CERK) to form ceramide 1-phosphate (C1P), used as a substrate by SMS to produce SM and diacylglycerol (DAG), modified with covalently linked sugar moieties to create glycosphingolipids, or hydrolyzed by ceramidases (CDase), creating Sph and a free fatty acid. Like dhSph, Sph can be phosphorylated by Sphks, producing sphingosine 1-phosphate (S1P), which can be dephosphorylated by S1P phosphatase (SPP1 and 2) or lipid phosphate phosphatase 3 (LPP3) back to Sph or terminally degraded by S1P lyase to phosphoethanolamine to produce phospholipids and hexadecenal and subsequently provide palmitoyl-CoA. In the salvage pathway, Cer is produced from SM hydrolysis by sphingomyelinases (SMase).

S1P is the product of kinase activity on Sph, which is produced by ceramidase hydrolysis of ceramides (**Figure 1**) (23, 24). *De novo* production of Cer begins with serine palmitoyltransferase (SPT)-mediated condensation of serine and palmitoyl-coenzyme A (CoA) and reduction of that product to generate dihydroSph (dhSph), which is then acylated to form dihydroCer (dhCer) (25, 26). Desaturation of dhCer generates Cer, where branching of the sphingolipid metabolic pathways rapidly multiplies, leading to production of phosphorylated forms (ceramide 1-phosphate, C1P) or sphingomyelin (SM) and diacylglycerol (DAG) production by sphingomyelin synthases (SMS) (27–30). If alpha-hydroxy (αOH)-fatty acyl-CoAs are utilized by dihydroceramide synthases (dhCers) rather than non-hydroxylated fatty acyl-CoAs, α-hydroxylated Cer (αOHCer) are synthesized (31). Changes in subcellular location or availability of enzymes and/or substrates also add dimension along this pathway. For instance, targeting of sphingosine kinase 1 (Sphk1) to the endoplasmic reticulum (ER) results in increased phosphorylation of dhSph to dihydroS1P (dhS1P) (32).

While cellular sources of blood and lymph S1P have recently been more clearly defined and the enzymes involved are now known, the source of the metabolic precursors, the mechanisms of S1P chaperone choice and loading, and their impact on metabolic flux in the SPL pathways remain to be fully characterized. Since altering abundance of one SPL family member can affect dramatic changes in both direct and distant metabolic precursors and products, it is reasonable to hypothesize that altering S1P chaperone capacity may trigger a response by S1P precursor molecules (33–35).

The paucity of studies reporting SPL concentrations in the lymph and the intriguing lack of effect on lymph S1P in the absence of ApoM led us to determine concentrations of an expanded panel of SPL species in blood and lymph from WT, *Apom^-/-^* (KO), and *APOM^Tg^* (Tg) mice, which constitutively express a human APOM transgene (1). The goal was to create the first murine lymph and corresponding blood plasma SPL profiles while using genetic models to examine how ApoM concentration affects the circulating sphingolipidome. This will provide a starting point for future investigations of how these two pools of circulating SPLs may influence mammalian physiology.

## Materials and methods

### Animals

Animals were housed in a specific pathogen-free facility and provided food and water *ad libitum*. All animal protocols were approved by the IACUC of Weill Cornell Medicine. *Apom^-/-^* (KO) and *APOM^Tg^* (Tg) mice were crossed at least 9 generations to the C57BL/6J genetic background and were previously described (1, 36). KO mice were created by deletion of 39 bp of *Apom* exon 2. Tg mice have a 6817 bp insertion of the human APOM sequence. C57BL/6 mice purchased from The Jackson Laboratory were used as wild-type (WT) controls. Female and male mice 8-9 weeks old were used for experiments. Animals were provided Teklad global 18% protein rodent diet (TD2018) and water *ad libitum*. WT, KO, and Tg mice from which suitable blood and lymph samples had been obtained were randomly selected from four different experiments to be included in this study.

### Blood and lymph plasma collection

Mice were euthanized with CO_2_. 800 µl to 1 mL of blood was recovered via terminal cardiac puncture and collected in tubes containing 35 µl 0.1M EDTA (EDTA_f_ = 3.5 - 4.375 mM). Samples were immediately mixed by inversion before being placed on ice. 500 µl whole blood was removed to a new tube and centrifuged at 2,000 x *g* for 15 minutes at 4°C. Plasma was removed and stored at -80°C until extraction and analysis. Lymph was recovered from the thoracic duct as previously described and collected in 5 µl acid citrate-dextrose (7, 37). The entire sample was centrifuged at 2,000 x *g* for 15 minutes to isolate plasma. Total lymph plasma volume of each sample was determined before being removed to a new tube and stored at -80°C. If evidence of blood contamination was observed in the lymph (pink color, sedimented erythrocytes upon centrifugation), samples from that animal were not used in this study.

### Blood and lymph sample preparation

Total lymph plasmas from five mice were pooled to create a sample volume suitable for lipidomic analysis. 10 µl of this lymph/anticoagulant mix was transferred to a new tube and 90 µl H_2_O added before storage at -80°C for shipment on dry ice to the Medical School of South Carolina (MUSC) Lipidomics Core for processing and analyses. For each genotype, two tubes of pooled lymph samples were created. 20 µl of blood plasma from the same five animals was pooled to yield a matching blood plasma sample for lipidomic analyses. A previous study found that concentrations of plasma (non-glyco) sphingolipids were not significantly affected by two freeze-thaw cycles (38).

### Lipid quantitation and calculations

SPL were extracted from blood or lymph plasma by single phase ethyl acetate:isopropanol:water process and quantitated by electrospray ionization-tandem mass spectrometry (ESI-MS/MS) as described in detail elsewhere (39–41). Briefly, samples were brought to 2 mL with serum-free medium before addition of 50 µl internal standards in methanol (1 µM Sph (d17:1), S1P (d17:1), 13C_16_-Cer, 17C_16_-Cer, 17C_24:1_-Cer, 18C_17_-Cer, and 18C_17_-dhCer, and 5µM 18C_8_-SM, 18C_12_-SM, and 18C_17_-SM), which were obtained primarily from the synthetic unit of the MUSC Lipidomics Shared Resource facility, and from commercially available sources, Avanti Polar Lipids Inc. (Alabaster, AL) and Matreya LLC (Pleasant Gap, PA), with purity of 98% or greater. Samples were then extracted with isopropyl:ethyl acetate (15:85, v:v) and divided for further processing specific for sphingoid bases/ceramides or SM/DAG species. At the time of our analyses, no authentic standards were available for some lipids of interest, particularly the dhSM species. Therefore, calculations for retention times (RT) and quantitation of these SPL species utilized surrogate calibration curves generated from the most similar SPL counterpart available. For each species, the standard used to create the calibration curve and mean retention times (min) are listed in **Supplementary Table 1**.

High lymph dilution with anticoagulant and variation in volumes obtained were unavoidable consequences of sample collection. To determine the molar concentration in lymph, 5 µl was subtracted from the total volume measured for each animal at the time of lymph plasma isolation to account for anticoagulant volume, giving the actual volume of plasma in that sample. Total volumes of lymph plasma + acid citrate dextrose and calculated total volumes of actual lymph plasma for each pooled sample were then used to determine molar concentrations and based on the resultant pmol/total sample derived from ESI-MS/MS analyses.

### Statistical analyses

Statistical analyses were performed using GraphPad Prism software v7.0-9.3. Two-tailed Student’s *t*-test was used for direct comparison of KO or Tg means to the WT mean. Several factors led us to not adjust for multiple comparisons. The corrected α (familywise error rate) for multiple comparisons were based upon the number of lipid species analyzed within a given family (e.g., 13 Cer versus 20 DAG versus 12 αOHCer species). Thus, the likelihood of a comparison with *p* ≤ 0.05 could vary dramatically based upon how we grouped lipid species for analysis (e.g., more likely to find a difference in αOHCer or Cer versus DAG). Because of the exploratory nature of this study and the low *n*, we have set α = 0.05 per comparison and created tables containing the nM values of each species within each individual sample and their means (42). Equal variances were assumed within blood or lymph; however, for any direct comparisons between blood and lymph, Welch’s *t*-test was used.

## Results

### Effect of ApoM genotype on lymph volume, total SPL mass, and S1P

**Figure 2:**
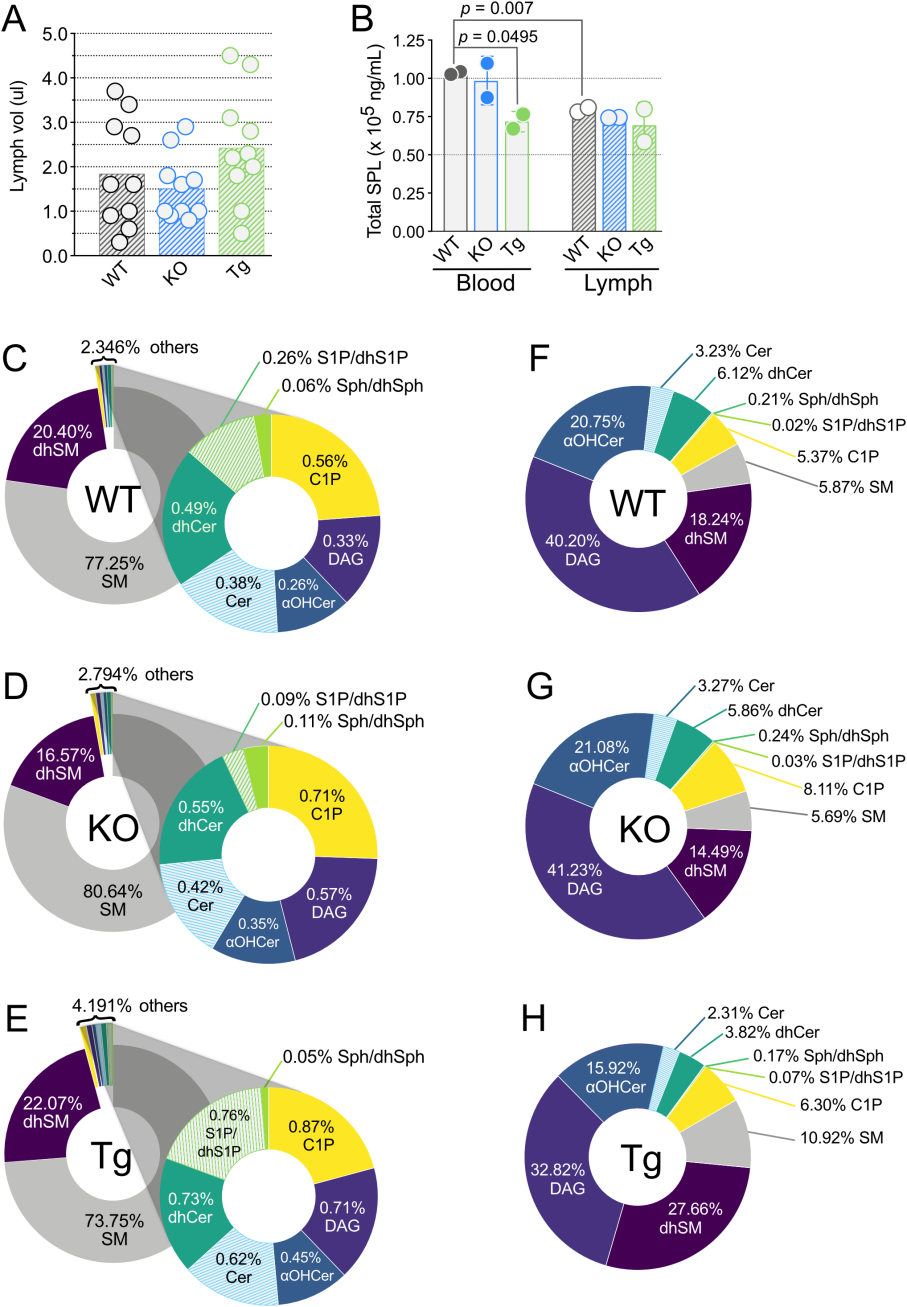
Lymph volume, total sphingolipid concentrations, and percentages of individual sphingolipid classes in wild-type (WT), *Apom^-/-^* (KO), and *APOM* transgenic mice (Tg). **(A)** Volume of lymph (µl) obtained from individual WT (black), KO (blue), and Tg (green) animals before pooling for analyses. Bars represent means and circles represent values from individual animals. **(B)** Total sphingolipid concentrations (ng/mL) in blood (open bars) and lymph (hashed bars). Bars represent means ± SD and circles represent values from separate pooled samples. **(C–E)** Percentage of each sphingolipid class in blood. **(F–H)** Percentage of each sphingolipid class in lymph. αOHCer, alpha-hydroxyceramide; Cer, ceramide; C1P, ceramide 1-phosphate; DAG, diacylglycerol; dhCer, dihydroceramide; dhSM, dihydrosphingomyelin; dhSph, dihydrosphingosine; dhS1P, dihydrosphingosine 1-phosphate; SM, sphingomyelin; Sph, sphingosine; S1P, sphingosine 1-phosphate.

Whole blood and lymph plasmas (referred to as “blood” and “lymph” unless otherwise stated) were collected from WT, KO, and Tg mice. KO mice have increased triacylglyceride (TAG; also known as triglyceride) metabolism and conversely, mice with the human APOM transgene exhibit decreased TAG clearance from circulation (43, 44). Altered TAG concentrations were evident in the lymph opacity: Tg mice had milky white lymph and an easily identifiable thoracic duct, whereas KO mice had dramatically reduced lymph opacity, increasing the difficulty of identifying the thoracic duct and obtaining samples not contaminated by blood (data not shown). Exclusion of subjects based on blood contamination of lymph led to a slightly increased minimum lymph volume obtained in KO versus Tg or WT (**Figure 2A**). However, the lymph volume means and ranges were similar for all three genotypes.

100 unique SPL species representing eight SPL and related family groups were assayed for: sphingomyelins (SM), dihydrosphingomyelins (dhSM), diacylglycerols (DAG), ceramides (Cer), dihydroceramides (dhCer), alpha-hydroxy ceramides (αOHCer), ceramide 1-phosphate (C1P), sphingosine/dihydrosphingosine (Sph/dhSph), and sphingosine 1-phosphate/dihydrosphingosine 1-phosphate (S1P/dhS1P) (**Supplementary Table 1**). In WT mice, 96 distinct SPL species were found in blood and 88 in lymph. 96 and 97 SPL were quantitated in blood of KO and Tg mice, respectively, and 90 (KO) or 89 (Tg) SPL species in lymph. Overall, of the 100 unique species targeted, 97 SPL species were identified in blood and 94 in lymph, regardless of ApoM genotype. When compared by total SPL mass, there was significantly less SPL per mL in WT lymph versus blood (**Figure 2B**). Although the number of SPL species in blood was similar between all ApoM genotypes, blood from Tg mice had significantly lower total SPL mass compared to WT control. Lymph total SPL mass did not differ between ApoM genotypes (**Figure 2B**).

### SM is the predominant SPL in murine blood

Mass percentage calculations were performed to determine the contribution of each lipid family to the total SPL composition of blood and lymph (**Figures 2C–H**, respectively) in mice of different ApoM genotypes. As previously reported for human blood plasma, SM predominated in mouse blood plasma, regardless of ApoM expression (**Figures 2C–E**) (38). dhSM was the second most abundant and all other species combined for 2.346% of the WT total blood SPL mass. S1P/dhS1P were only 0.26% of the SPL mass percentage of WT blood but were four times the mass of their precursors Sph/dhSph (0.06%). dhSM and dhSph can serve as metabolic precursors for dhCer, which at 0.49% was almost twice as abundant as S1P/dhS1P. Cer, the desaturation product of dhCer, was less abundant at 0.38%, but the phosphorylated forms, C1P, were the third highest SPL in total mass percentage at 0.56%. DAG, a product of the conversion of Cer to SM, was only 0.33%. Surprisingly, αOHCer, an alternative metabolite of dhSph when CerS incorporates an αOH-fatty acyl-CoA instead of a non-hydroxylated fatty acyl-CoA, constituted 0.26% of WT blood total SPL mass, equivalent to S1P/dhS1P. To our knowledge, this is the first report of αOHCer in circulation.

We then compared these values to those obtained for blood from KO and Tg animals (**Figures 2D, E**). S1P/dhS1P dropped to 0.09% in KO and rose to 0.76% in Tg, approximating the expected ~65% decrease and ~300% increase, respectively (2, 7). Although S1P contributes less than half a percent to total SPL mass in blood, the interdependency of SPL metabolic pathways suggests compensatory changes in other low mass percentage lipids could be anticipated in both KO and Tg blood. The mass percentage of the S1P/dhS1P precursors Sph/dhSph increased to 0.11% in KO blood but were unchanged in Tg (0.05%). Mass percentages of Cer, dhCer, αOH-Cer, C1P, and DAG all increased in KO blood; however, they also increased in the blood of Tg animals. Thus, the total contribution of the minor blood SPL species increased from 2.346% in WT to 2.794% in KO and almost doubled to 4.191% in Tg mice. The greatest losses and gains in blood SPL mass percentage were seen in the two most abundant families. KO blood had 3.39% increased SM, and 3.83% decreased dhSM, while the inverse was observed in Tg blood: dhSM increased by 1.66% and SM decreased by 3.51%. These differences indicate that the presence or absence of ApoM protein shifts the mass percentages of most blood SPL by greater degrees than expected by simple subtraction or addition of its ligands.

### αOHCer is the predominant SPL in murine lymph

The detailed SPL composition of lymph is largely unknown. DAG was the major lipid species measured in WT lymph, constituting 40.20% mass percentage compared to the SPL families (**Figure 2F**). Measurements of αOHCer species again yielded surprising results, since they contributed the greatest SPL mass percentage in WT lymph. At 20.75%, the αOHCer were almost equal to the mass percentage of all other SPL species combined, minus dhSM. To our knowledge, this is also the first report of αOHCer in the lymph. dhSM were again the second greatest contributor to SPL mass percentage at 18.24%, similar to its blood SPL percentage. The dhSM product, dhCer was next in percent abundance at 6.12%. SM contributed only 5.87% SPL mass of lymph, less than one tenth their contribution to blood mass percentage. C1P were slightly less than SM (5.37%) and unphosphorylated Cer were 3.23%. Sph/dhSph were only 0.21% and S1P/dhS1P a minuscule 0.02% of the SPL mass percentage in WT lymph.

Many of the changes in SPL mass percentage of KO versus WT lymph were less dramatic than those seen in blood (**Figure 2G**). Loss of ApoM did not affect lymph mass percentage of S1P/dhS1P, and Sph/dhSph only slightly increased to 0.24% from 0.21%. The KO mass percentages of Cer, dhCer, SM, and αOHCer differed from their WT values by less than 0.5% and the DAG increased by 1% to 41.23%. C1P were the only SPL with a large percentage gain in KO lymph, 8.11% from 5.37% in WT, and dhSM the only sizeable loss, to 14.49% from 18.24% in WT lymph. Thus, loss of ApoM had a greater influence on the percent contribution of individual SPL families to total mass in blood compared to lymph.

Changes in SPL mass percentages compared to WT lymph were more pronounced in Tg lymph (**Figure 2H**). Although still small compared to other species, the percent S1P/dhS1P was three times greater in Tg lymph (0.07% versus 0.02% in WT) while precursors Sph/dhSph decreased by only 0.04% (from 0.21% to 0.17%). SM increased to 10.92%, almost doubling the SM contribution in WT. dhSM increased by 1.5 times to 27.66%, replacing αOHCer species as the most abundant SPL by mass percentage in Tg lymph. Although the change in dhCer was only 2.2%, it was a 40% decrease of the contribution in WT lymph. Cer also decreased in Tg by 0.92% while C1P increased by about the same percent, 0.93%. DAGs contributed 7.3% less to the mass percentage of Tg lymph compared to WT.

**Figure 3:**
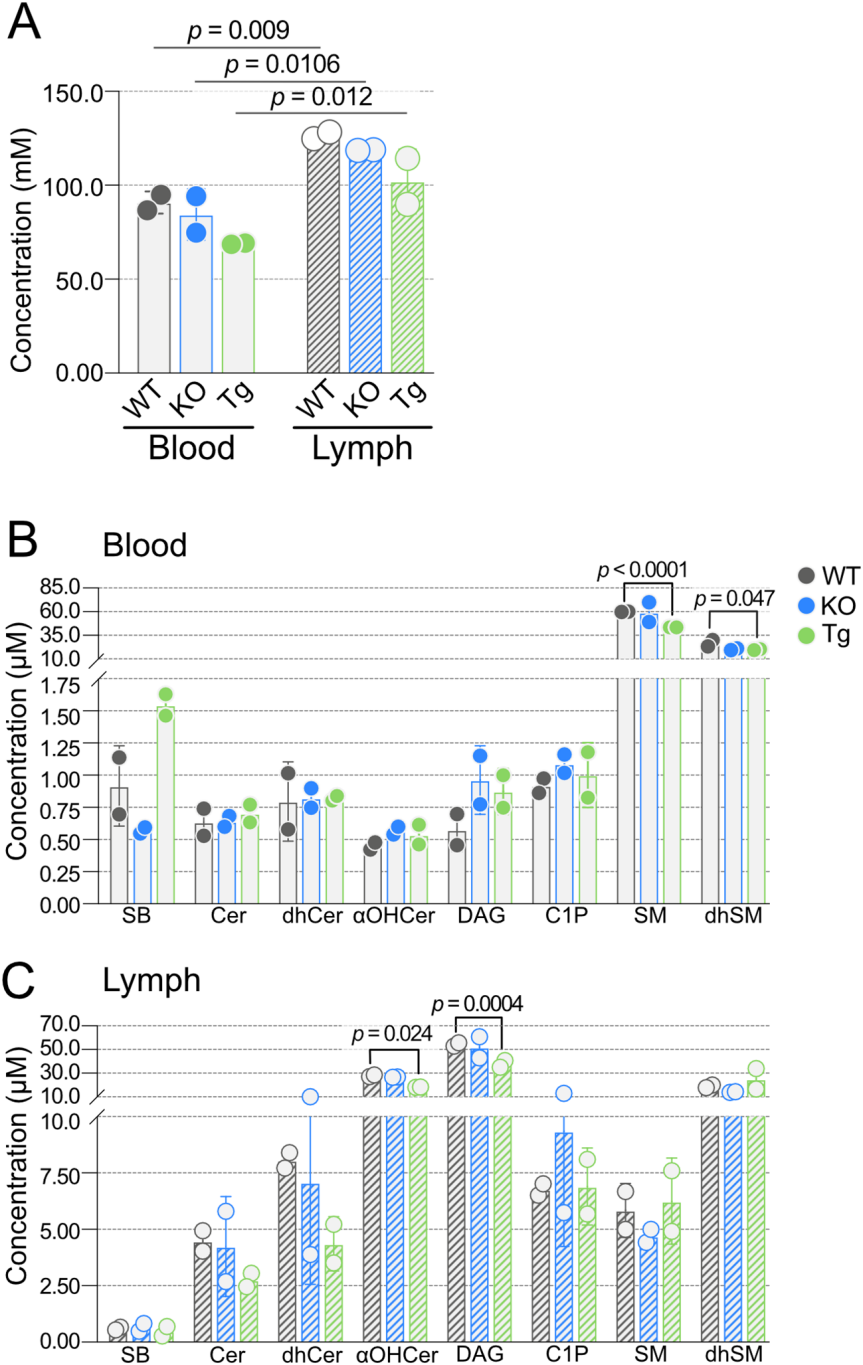
Concentrations of blood and lymph sphingolipid classes in wild-type (WT), *Apom^-/-^* (KO), and *APOM* transgenic (Tg) animals. **(A)** Combined concentrations (mM) of all sphingolipid species detected in blood (open bars) and lymph (hashed bars) of WT (black), KO (blue), and Tg (green) animals. **(B)** Concentrations (µM) of individual sphingolipid classes in blood. **(C)** Concentrations (µM) of individual sphingolipid classes in lymph. Bars represent means ± SD and circles represent values from separate pooled samples. αOHCer, alpha-hydroxyceramide; Cer, ceramide; C1P, ceramide 1-phosphate; DAG, diacylglycerol; dhCer, dihydroceramide; dhSM, dihydrosphingomyelin; SM, sphingomyelin; SB, sphingoid bases.

We next considered molar SPL concentrations in blood and lymph. Within the two tissues, there was no difference in total SPL molar concentrations between KO or Tg compared to WT mice (**Figure 3A**). However, total molar SPL concentrations were significantly increased in lymph compared to blood within each genotype. With the exception of SM and dhSMN (**Figure 3B**), most of the SPL families in blood were found in high nanomolar to low micromolar concentrations (less than 1.5 µM). Concentrations of all SPL families were statistically similar between KO and WT blood; however, total SM and dhSM concentrations in Tg blood were significantly lower than WT. In contrast, all SPL groups except sphingoid bases (SB) Sph, dhSph, S1P, and dhS1P, were over 2.5 µM in lymph (**Figure 3C**). DAG and αOHCer were found in the highest concentrations. Similar to blood, total SPL concentrations in KO lymph did not differ from WT, whereas αOHCer and DAG were significantly lower in Tg compared to WT lymph.

### Effects of ApoM expression on sphingoid bases are tissue-specific

**Figure 4:**
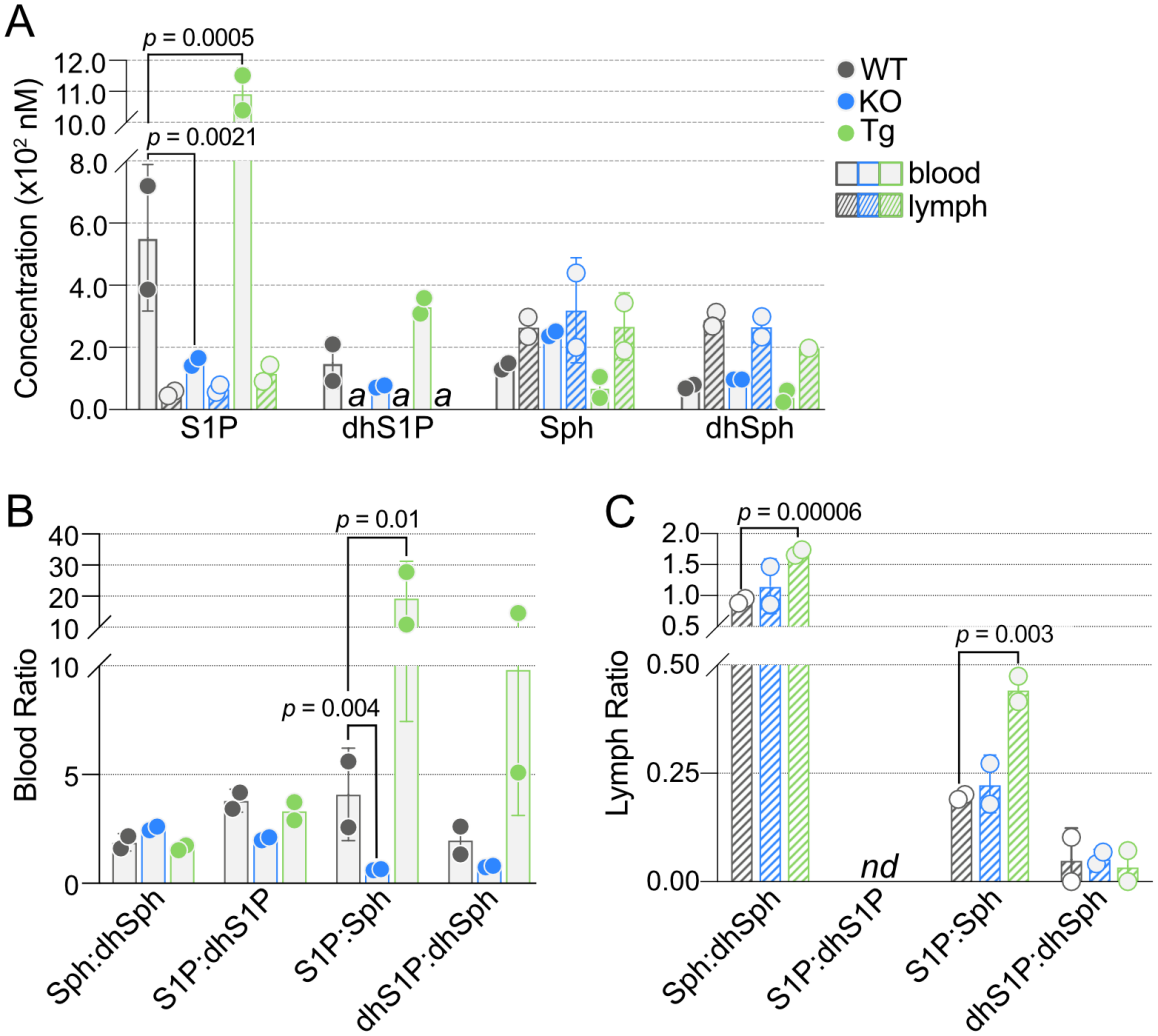
Quantification of blood and lymph sphingoid bases in wild-type (WT), *Apom^-/-^* (KO), and *APOM* transgenic (Tg) mice. **(A)** Concentrations (nM) of S1P, dhS1P, Sph, and dhSph in blood (open bars) and lymph (hashed bars) of WT (black), KO (blue), and Tg (green) animals. **(B)** Ratios of sphingoid bases in blood of WT, KO, and Tg animals. **(C)** Ratios of sphingoid bases in lymph of WT, KO, and Tg animals. Bars represent means ± SD and circles represent values from separate pooled samples. dhSph, dihydrosphingosine; dhS1P, dihydrosphingosine 1-phosphate; Sph, sphingosine; S1P, sphingosine 1-phosphate. (a): value is from a sample whose signal was one to two times that of the matrix blank; *nd*: at least one value used to compute the ratio is from a sample whose signal was below the detection limit and thus the ratio is undefined.

One of the primary roles of ApoM is the strong and specific binding of S1P in blood (22, 45). As expected, S1P concentrations were significantly lower in the blood of KO and higher in the blood of Tg mice compared to WT controls, and dhS1P showed similar trends (**Figure 4A**, **Supplementary Table 2**). While Sph trended higher in KO and lower in Tg blood, this did not reach significance. Analysis of lymph revealed no change in concentrations of S1P or the other sphingoid base, Sph, confirming our previous report (7).

The interconnectedness of the SPL pathways can make ratios a useful method for examining perturbations in various lipid species. In particular, the ratios of Sph and S1P to their fully saturated dihydro forms, dhSph or dhS1P, or the ratio of S1P to its parent molecule, Sph (or dhS1P to dhSph). S1P:Sph was significantly different in both KO and Tg, which correlated with the significant changes in S1P concentrations (**Figure 4B**). There was a similar trend in dhS1P:dhSph. Although blood S1P:dhS1P was decreased in KO compared to WT, it was not significant. Neither of these ratios in lymph were individually affected by ApoM genotype; however, lymph Sph:dhSph and S1P:Sph were significantly increased in Tg compared to WT (**Figure 4C**).

### Greater SPL species diversity in lymph versus blood

**Figure 5:**
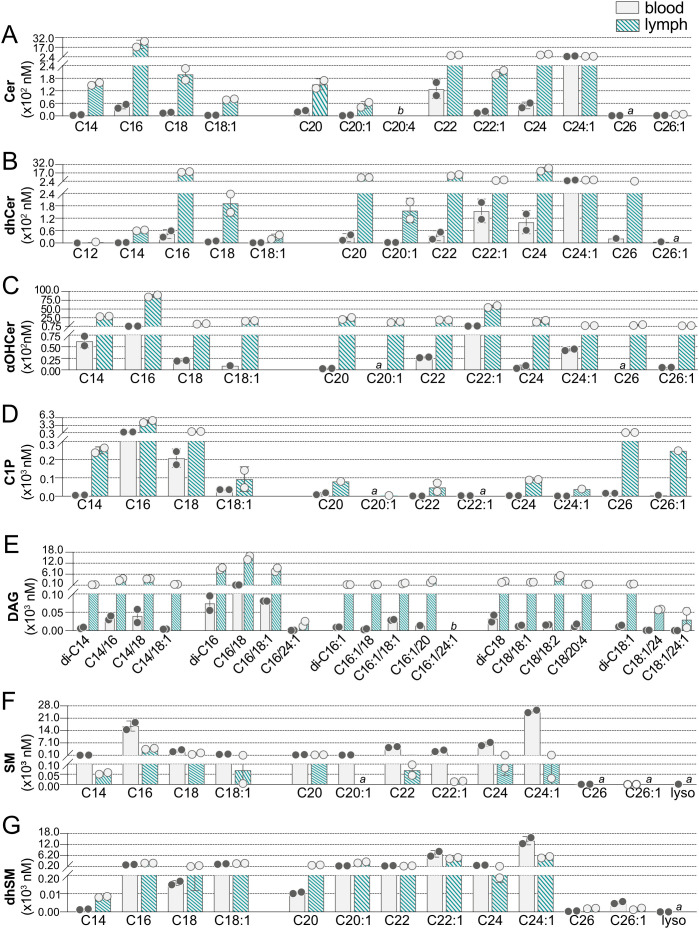
Concentrations of individual sphingolipid species in blood and lymph of wild-type (WT) mice. Concentrations (nM) of individual sphingolipid species were determined in blood (open bars) and lymph (hashed bars) of WT mice and are shown grouped according to sphingolipid class: **(A)** Cer (ceramides); **(B)** dhCer (dihydroceramides); **(C)** αOHCer (α-hydroxyceramides); **(D)** C1P (ceramide 1-phosphates); **(E)** DAG (diacylglycerols); **(F)** SM (sphingomyelins); **(G)** dhSM (dihydrosphingomyelins). Bars represent means ± SD and circles represent values from separate pooled samples. (a) value is from a sample whose signal was one to two times that of the matrix blank; (b) value is from a sample whose signal was below that of the matrix blank.

To create a full sphingolipidomics analysis comparing blood versus lymph lipid species and their concentrations, we next determined concentrations of individual Cer, dhCer, αOHCer, C1P, DAG, SM, and dhSM in blood and lymph of WT mice (**Figure 5**, **Supplementary Tables 3–9**). Similar to results from humans, C22:0, C24:0, and C24:1 species had the greatest contribution to blood Cer (**Figure 5A**, **Supplementary Table 3**). In addition to these species, C22:1 and particularly C16:0 Cer were also at high concentrations in lymph with C16:0 at micromolar levels. The majority of blood dhCer (**Figure 5B**, **Supplementary Table 4**) was C24:1, whereas long chain and very long chain (>20 carbons) dhCer were present in lymph at high nanomolar to low micromolar concentrations. Long chain αOHCer (**Figure 5C**, **Supplementary Table 5**) species C14 and C16 were present in blood, but C22:1 was the most abundant at 162.13 nM. Surprisingly, all αOHCer species assayed for were detected in lymph, ranging from 275.30 nM (C26:1) to 8.753 µM (C16:0). C1P (**Figure 5D**, **Supplementary Table 6**) in blood was primarily restricted to long chain species C16 and C18. Lymph contained primarily C16 and C18 C1P, but also moderate concentrations of C14 (250.96 nM), C26 (423.07 nM), and C26:1 (248.3 nM) C1P. Blood DAG (**Figure 5E**, **Supplementary Table 7**) consisted primarily of DAG species with C16 in the *sn-*1 position (di-C16, C16/18, C16/18:1), which were also the most abundant species in lymph. Except for C16:1/24:1, most DAG species targeted were detected in lymph, and half were at micromolar concentrations. Overall, compared to WT blood, WT lymph had a greater variety of high-abundance Cer, dhCer, αOHCer, C1P, and DAG.

While many of the lipid species targeted were not found in blood but were present in lymph, SM showed the opposite trend (**Figure 5F**, **Supplementary Table 8**). SM species of C14 through C24:1 were all found at medium to high nanomolar concentrations in blood, with C24:1 and C16 being the most abundant. At less than one-fifth the concentration in blood, C16 was also the most abundant SM species in lymph. Although C20:1 SM was detected in blood, it was not found in lymph and the 26 carbon SM species were below the detection limit in both blood and lymph. The dhSM (**Figure 5G**, **Supplementary Table 9**) were the only SPL family with the majority of species found in both blood and lymph at similar concentrations.

### ApoM expression alters SPL levels in both blood and lymph

**Figure 6:**
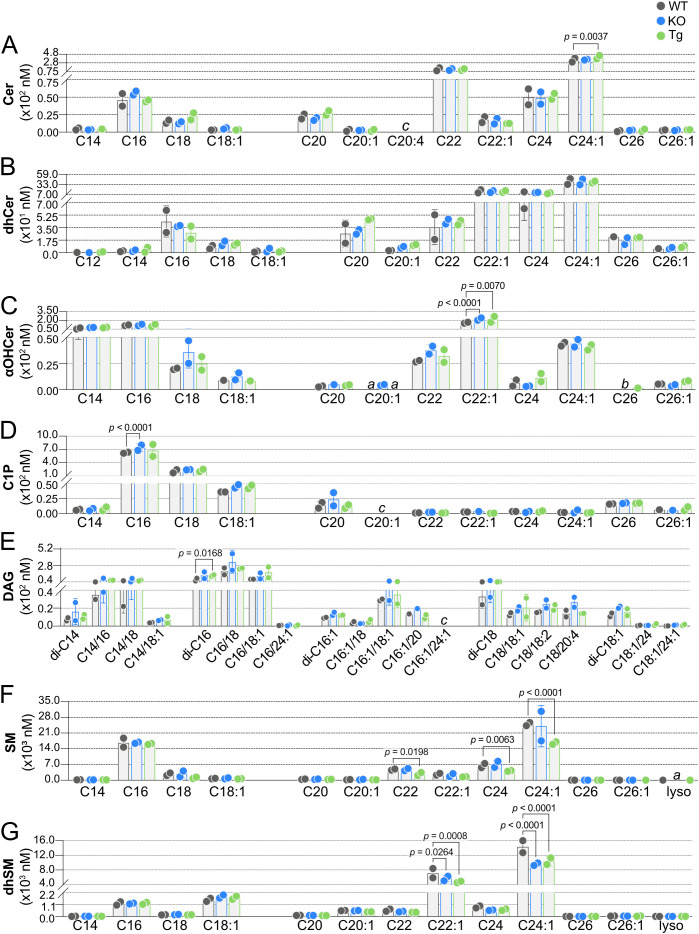
Effect of ApoM expression on individual sphingolipid species concentrations in blood from wild-type (WT), *Apom^-/-^* (KO), and *APOM* transgenic (Tg) mice. Concentrations (nM) of individual sphingolipid species were determined in blood of WT (black), KO (blue), and Tg (green) animals and are shown grouped according to sphingolipid class: **(A)** Cer (ceramides); **(B)** dhCer (dihydroceramides); **(C)** αOHCer (α-hydroxyceramides); **(D)** C1P (ceramide 1-phosphate); **(E)** DAG (diacylglycerols); **(F)** SM (sphingomyelins); **(G)** dhSM (dihydrosphingomyelins). Bars represent means ± SD and circles represent values from separate pooled samples. Statistical differences detected between WT versus KO or Tg samples are indicated with the calculated *p* value. (a) value is from a sample whose signal was one to two times that of the matrix blank; (b) value is from a sample whose signal was below that of the matrix blank. (c) value is from a sample whose signal was below the detection limit.

Having established baseline WT concentrations for SPL species in blood and lymph, the impact of ApoM knockout or overexpression on these species was determined. In KO blood, no Cer were altered, and only a single Cer, C24:1 Cer, was significantly affected by Tg expression (**Figure 6A**). dhCer was the only SPL family assayed that was not significantly affected in blood by altered ApoM expression (**Figure 6B**). αOHCer C22:1, already the highest concentration blood αOHCer, was significantly further increased in both KO and Tg blood compared to WT (**Figure 6C**). Similar to precursor molecule Cer, blood concentrations of C1P species (**Figure 6D**) were mostly unaffected, with the exception of increased C16 C1P in KO blood. A single DAG, di-C16 (**Figure 6E**), was significantly changed in Tg blood with no changes in KO. SM and dhSM (**Figures 6F, G**, respectively) were unique as the only SPL families with species significantly decreased in blood as a result of altered ApoM expression and also had the most species affected. While ApoM KO did not affect blood SM concentrations, C22, C24, and C24:1 SM were significantly decreased in Tg compared to WT blood. C22:1 and C24:1 dhSM were significantly decreased in Tg as well as KO blood. With the exception of SM C22 and C24, the blood SPL species significantly changed as a result of ApoM expression were the most abundant species in their respective families.

**Figure 7:**
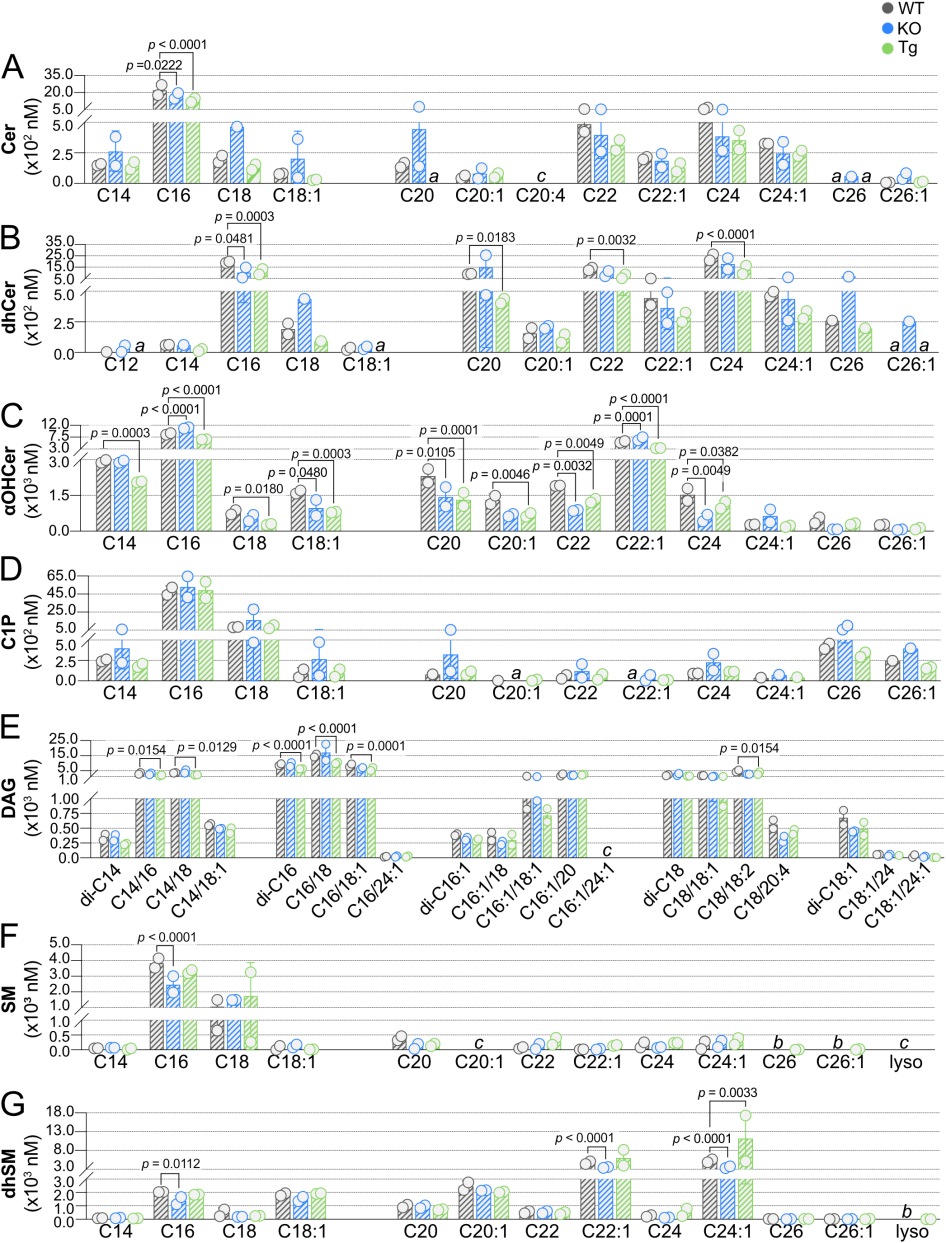
Effect of ApoM expression on individual sphingolipid species concentrations in lymph from wild-type (WT), *Apom^-/-^* (KO), and *APOM* transgenic (Tg) mice. Concentrations (nM) of individual sphingolipid species were determined in lymph of WT (black), KO (blue), and Tg (green) animals and are shown grouped according to sphingolipid class: **(A)** Cer (ceramides); **(B)** dhCer (dihydroceramides); **(C)** αOHCer (α-hydroxyceramides); **(D)** C1P (ceramide 1-phosphate); **(E)** DAG (diacylglycerols); **(F)** SM (sphingomyelins); **(G)** dhSM (dihydrosphingomyelins). Bars represent means ± SD and circles represent values from separate pooled samples. Statistical differences detected between WT versus KO or Tg samples are indicated with the calculated *p* value. (a) value is from a sample whose signal was one to two times that of the matrix blank; (b) value is from a sample whose signal was below that of the matrix blank. (c) value is from a sample whose signal was below the detection limit.

We next compared concentrations of SPL species in lymph from KO or Tg mice to concentrations in WT animals, where the influence of ApoM expression was more evident (**Figure 7**). Again, only a single Cer species was significantly affected by ApoM expression, Cer C16, decreased in both KO and Tg lymph (**Figure 7A**). Whereas blood dhCer had been unaffected, lymph concentrations of multiple dhCer species were significantly affected by ApoM expression: C20, C22, and C24 were decreased in Tg, and C16 decreased in both KO and Tg lymph (**Figure 7B**). The most affected by ApoM expression were lymph αOHCer: nine of 12 species were significantly different from WT (**Figure 7C**). All significantly different αOHCer species in Tg lymph (C14, C16, C18, C18:1, C20, C20:1, C22, C22:1, and C24) were decreased compared to WT controls. αOHCer C18:1, C20, C22, and C24 were also significantly decreased in KO. However, the two most abundant αOHCer species in WT lymph, C16 and C22:1, were significantly increased in lymph from KO mice (*p* < 0.0001 and *p* = 0.0001, respectively). Although lymph C1P concentrations were higher than those in blood, there was also greater variability within KO and Tg samples, and no significant differences from WT lymph C1P were detected (**Figure 7D**). As in blood, lymph DAG di-C16 was significantly affected in Tg animals, but decreased rather than increased compared to WT. Tg lymph also had significant decreases in DAG species C14/16, C14/18, C16/18, C16/18:1, and C18/18:2 (**Figure 7E**). No differences were seen in KO lymph DAG species. SM C16 was the sole lymph SM species affected and was decreased in KO compared to WT (**Figure 7F**). Lastly, KO lymph had significantly decreased concentrations of dhSM C16, C22:1, and C24:1, whereas C24:1 was significantly increased in lymph from Tg animals (**Figure 7G**). Overall, more than twice as many SPL and DAG species were significantly affected in the lymph versus blood by either KO or Tg expression of ApoM (9 blood species versus 24 lymph species).

**Figure 8:**
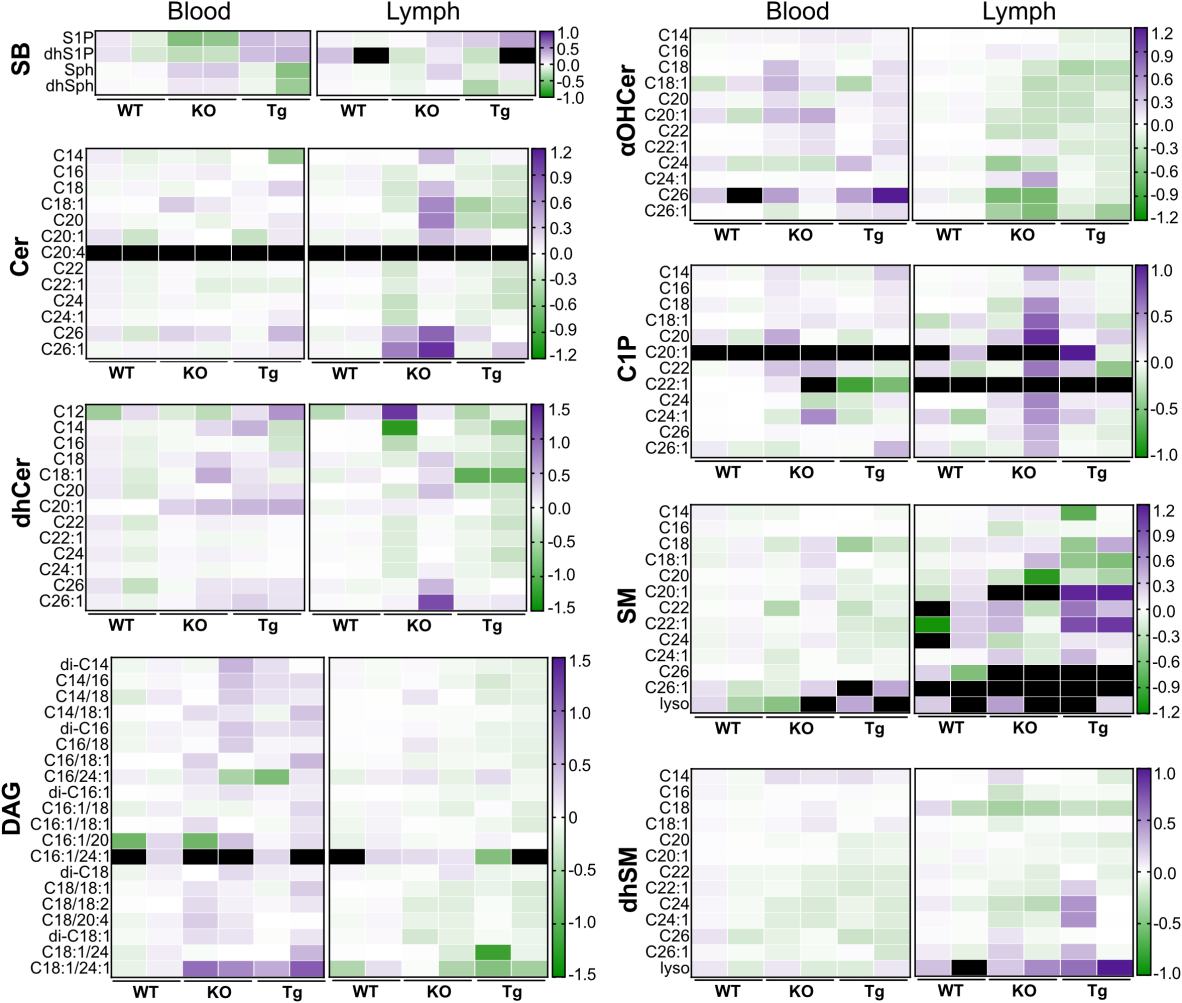
Heat map comparing concentrations of circulating lipid species in wild-type (WT), *Apom^-/-^* (KO), and *APOM* transgenic (Tg) mice. Concentration values shown in **Figure 6** (blood) and **Figure 7** (lymph) are normalized to WT values by dividing the concentration of each individual sample by the WT mean, followed by a log 10 transform. αOHCer, alpha-hydroxyceramide; Cer, ceramide; C1P, ceramide 1-phosphate; DAG, diacylglycerol; dhCer, dihydroceramide; dhSM, dihydrosphingomyelin; dhSph, dihydrosphingosine; dhS1P, dihydrosphingosine 1-phosphate; SB, sphingoid base; SM, sphingomyelin.

Concentration variability within genotypes or between tissues can be observed in **Figure 8**, a heat map summary of the data shown in **Figures 4**-**7**. When examined in this manner, it is more apparent when some species show higher variability whereas others are very similar (e.g., blood DAG C16:1/20 versus blood Sph species, respectively).

### Molar ratios of individual SPL species in WT blood:lymph

**Figure 9:**
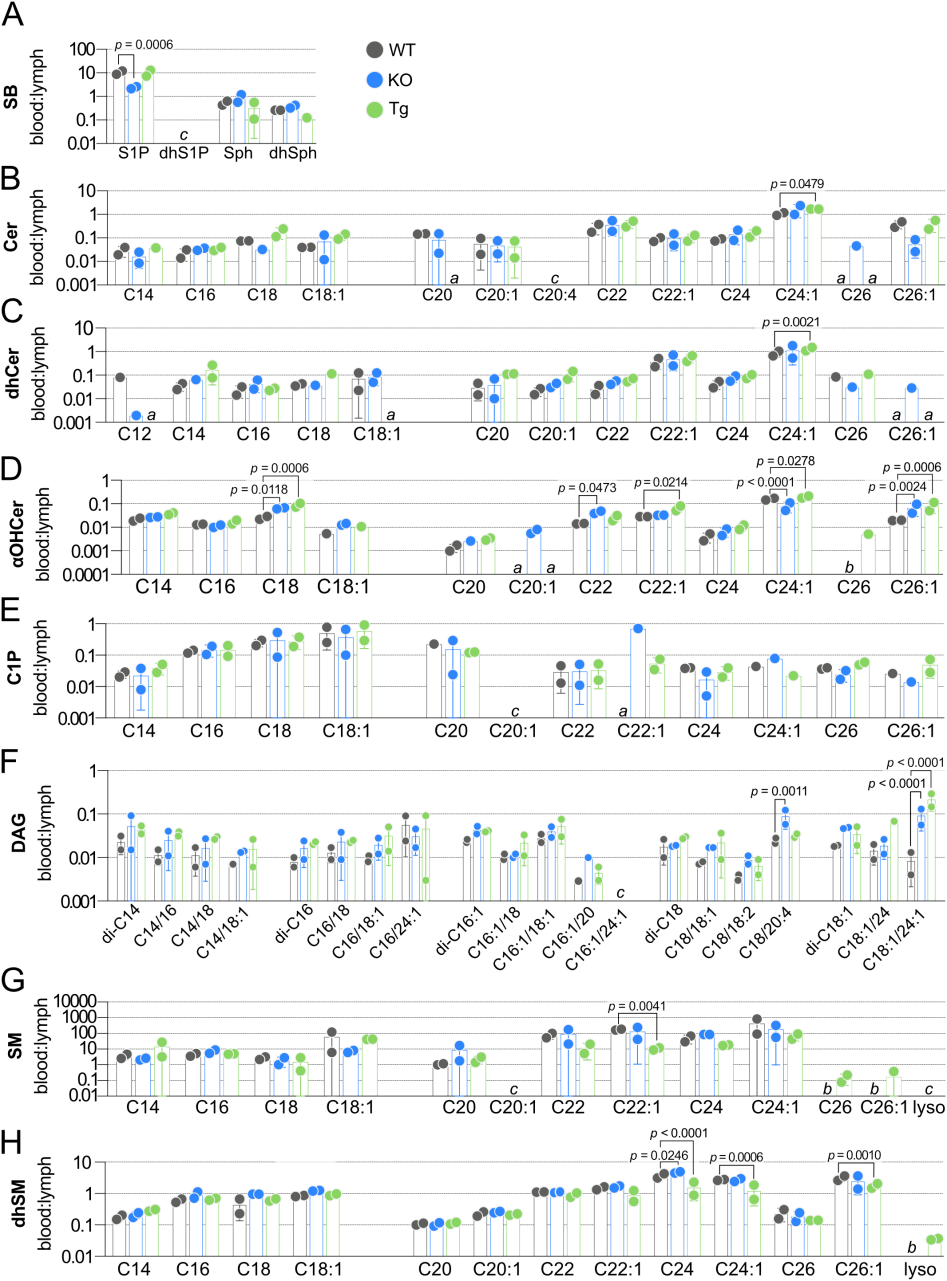
Effect of ApoM expression on blood:lymph ratios of individual sphingolipid species in wild-type (WT), *Apom^-/-^* (KO), and *APOM* transgenic mice (Tg), grouped according to sphingolipid class. Ratios of individual sphingolipid species were determined in samples from WT (black), KO (blue), and Tg (green) animals and are shown grouped according to sphingolipid class: **(A)** SB (sphingoid bases); **(B)** Cer (ceramides); **(C)** dhCer (dihydroceramides); **(D)** αOHCer (α-hydroxyceramides); **(E)** C1P (ceramide 1-phosphates); **(F)** DAG (diacylglycerols); **(G)** SM (sphingomyelins); **(H)** dhSM (dihydrosphingomyelins). Bars represent means ± SD and circles represent values from separate pooled samples. Statistical differences detected between WT versus KO or Tg samples are indicated with the calculated *p* value. (a) at least one value used to compute the ratio is from a sample whose signal was one to two times that of the matrix blank. (b) at least one value used to compute the ratio is from a sample whose signal was below that of the matrix blank. (c) at least one value used to compute the ratio is from a sample whose signal was below the detection limit and thus the ratio is undefined.

Although lymph plasma originates from multiple sites in the body, the base of lymph is primarily derived from blood plasma (15). To investigate how the two pools might influence each other, we next determined the blood:lymph molar ratios of individual SPL (**Figure 9**, **Supplementary Tables 10–17**). Our initial targets were the SB: S1P, dhS1P, Sph, and dhSph (**Figure 9A**, **Supplementary Table 10**). While S1P was ten times higher in WT blood versus lymph, the unphosphorylated forms Sph and dhSph were found at approximately equal concentrations in blood and lymph. The decrease in KO blood S1P coupled with unchanged lymph S1P concentrations dramatically decreased the blood:lymph S1P ratio; however, ratios of Sph and dhSph were not affected. The low molar ratios of Cer (**Figure 9B**, **Supplementary Table 11**) and dhCer (**Figure 9C**, **Supplementary Table 12**) species emphasize their much higher concentrations in lymph versus blood. Only Cer C24:1 and dhCer C24:1 had blood:lymph greater than 1.0, and were also the only significantly affected Cer or dhCer species, both significantly increased in Tg animals.

The low blood:lymph ratio of all αOHCers species reflects their micromolar contributions to total lymph SPL versus low nanomolar concentrations in blood (**Figure 9D**, **Supplementary Table 13**). In Tg animals, significant increases in blood and decreases in lymph αOHCer C22:1 resulted in a significant change in the blood:lymph of this species. Significant increases in KO blood:lymph αOHCer C18 and C22 were driven by increases in blood and decreases in lymph concentrations. Blood:lymph αOHCer C18, C24:1, and C26:1 were significantly increased in Tg samples. KO also had increased blood:lymph C26:1 but decreased C24:1. All C1P species were higher in lymph than in blood and had ratios less than one largely unaffected by ApoM KO or Tg expression (**Figure 9E**, **Supplementary Table 14**). Although their concentrations in either blood or lymph were unchanged in KO and Tg samples, blood:lymph ratios of low abundance DAG C18/20:4 (KO) and C18:1/24:1 (KO and Tg) were significantly different from WT (**Figure 9F**, **Supplementary Table 15**). In contrast to the other SPL species besides S1P, most of the detected SM species (**Figure 9G**, **Supplementary Table 16**) and a third of the dhSM species (**Figure 9H**, **Supplementary Table 17**) were higher in blood than lymph. SM C22:1 blood:lymph was decreased in Tg without significant changes in either blood or lymph concentrations. Similarly, blood:lymph dhSM changes in C24 (KO and Tg) and C26:1 (Tg) were not the result of changes in the individual compartments, whereas the decrease in Tg blood:lymph dhSM C24:1 was the result of decreased blood and increased lymph concentrations.

### SPL chain length and saturation in blood and lymph

**Figure 10:**
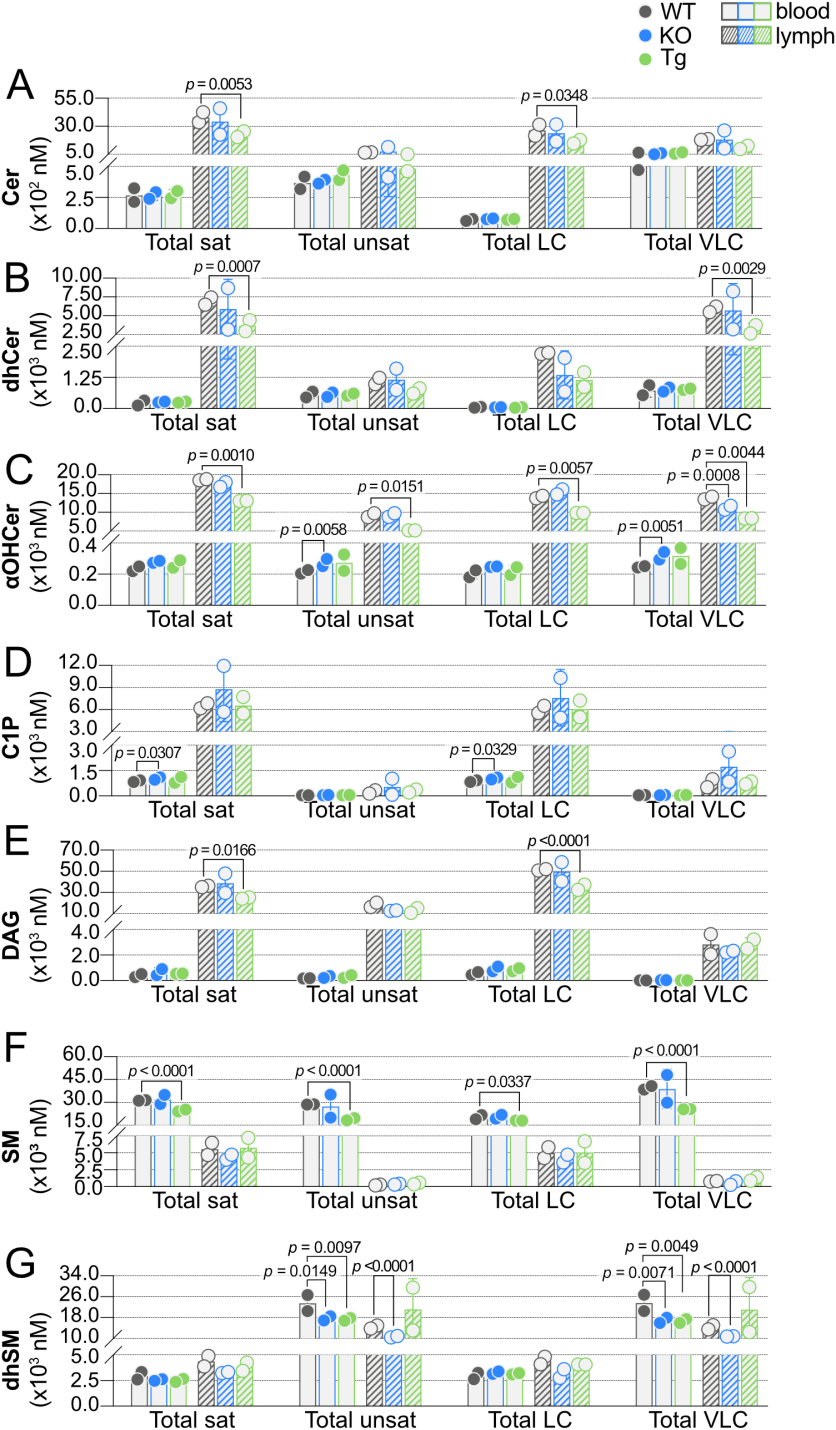
Effect of ApoM expression on carbon chain saturation and length. Total concentrations (nM) of saturated (sat), unsaturated (unsat), long chain (LC; ≤ 18 carbons), and very long chain (VLC; ≥ 20 carbons) sphingolipids were determined in blood (open bars) and lymph (hashed bars) of wild-type (WT), *Apom^-/-^* (KO), and *APOM* transgenic (Tg) mice. Results are shown grouped according to sphingolipid class: **(A)** Cer (ceramides); **(B)** dhCer (dihydroceramides); **(C)** αOHCer (α-hydroxyceramides); **(D)** C1P (ceramide 1-phosphates); **(E)** DAG (diacylglycerols); **(F)** SM (sphingomyelins); **(G)** dhSM (dihydrosphingomyelins). Bars represent means ± SD and circles represent values from separate pooled samples. Statistical differences detected between WT versus KO or Tg values are indicated with the calculated *p* value.

Carbon chain saturation and acyl chain length can dramatically affect SPL biological activities through altered binding and signaling properties or inducing biophysical changes in cell membranes (46, 47). Such differences have drawn interest as potential biomarkers in human diseases, leading us to further analyze our sphingolipidomics data on the basis of acyl chain saturation (saturated versus unsaturated) and carbon number (LC, 12-18 carbons, versus VLC, ≥ 20 carbons), shown in **Figure 10** (25, 48–52). Lymph concentrations of saturated Cer, dhCer, αOHCer, C1P, and DAG were 10-20 times concentrations in blood (**Figures 10A–E**). Curiously, total unsaturated Cer, dhCer, and C1P concentrations were similar between blood and lymph, whereas unsaturated αOHCer and DAG concentrations were similar to their saturated species and more than 10 times greater in lymph than blood. SM species were unique in that both saturated and unsaturated SM were much higher in blood than lymph (**Figure 10F**). dhSM species were also unique, with roughly equivalent concentrations of saturated and unsaturated species in blood compared to lymph (**Figure 10G**).

With regard to acyl chain length, most of the total LC concentrations mirrored the blood versus lymph patterns seen within their respective total saturated concentrations, while total VLC mirrored that of unsaturated species. For instance, total LC Cer, αOHCer, C1P, and DAG were much higher in lymph than blood, whereas LC SM were higher in blood than lymph and LC dhSM species in blood and lymph were similar. While total unsaturated dhCer species were similar between blood and lymph, total VLC dhCer lymph concentrations were approximately 10 times their concentration in blood, but total unsaturated dhCer were similar between blood and lymph.

Significant decreases in lymph saturated SPL were seen between Tg and WT for four of the seven SPL groups: Cer, dhCer, αOHCer, and DAG. C1P in blood was the only group of saturated lipids that differed significantly between KO and WT animals; however, unsaturated αOHCer and dhSM were significantly changed in KO blood. Unsaturated dhSM was also significantly decreased in KO lymph and Tg blood. These significant decreases in KO and Tg blood and lymph were mirrored in VLC dhSM. When the influence of ApoM genotype on chain length was considered, KO resulted in significant increases in blood LC C1P and VLC αOHCer and significant decreases in VLC lymph αOHCer. Tg expression resulted in significant decreases only, regardless of tissue or chain length. LC and VLC SM were both decreased in Tg blood, as were VLC dhSM species. Lymph LC Cer, αOHCer, and DAG and VLC dhCer were significantly decreased in Tg versus WT samples. Overall, a pattern emerged upon sorting SPL groups by chain length and saturation: when total saturated species were affected by genotype, total LC species were also significantly affected or trended in the same direction within the same tissue. Similarly, if unsaturated species were significantly different, the total VLC species were affected or displayed a similar trend.

## Discussion

There is an increasing appreciation of the importance of circulating lipids beyond HDL/low-density lipoprotein (LDL) as indicators of health and disease status. Members of the SPL family, while structurally simple relative to proteins, are powerful signaling molecules and critical to cellular infrastructure. Sphingolipidomics of blood plasma and serum have uncovered potential biomarkers of early-stage disease and novel drivers of pathology in numerous diseases including stroke, amyotrophic lateral sclerosis (ALS), Alzheimer’s disease, SLE, non-alcoholic fatty liver disease (NAFLD), and sepsis (38, 48, 53–61). Recently, blood SPL concentrations have been correlated with COVID-19 severity in humans and animal models: blood concentrations of S1P, total HDL, ApoM, and the ratio of ApoM- versus albumin-bound S1P may be some of the most reliable predictors of COVID-19 morbidity and mortality (13, 62, 63).

By comparison, the lymph is often neglected and lymph “omics” studies are much less common than those of blood despite its crucial roles in inflammation, immunity, and maintenance of homeostasis (15, 64–66). Difficult sample collection, particularly from animal models, is likely the greatest contributor to the lack of lymph characterization. The overall disinterest in lymph may also stem from the misconception that it is merely filtered blood plasma components rather than a unique mixture consisting of metabolic products drained from each organ as well as immune cells, tumor extracellular vesicles, intracellular components, and soluble signaling mediators (67–69). Reports of lymph lipid composition are typically focused on free cholesterol, cholesterol esters, or phospholipids, with a few notable exceptions: it was recently reported that high lymph oleic acid created an antioxidative environment compared to blood plasma and protected metastasizing melanoma cells from ferroptosis (70–72). Regulation of lymphocyte trafficking by S1P receptors and searches for tissue-specific S1P transporters have produced measurements of lymph S1P and Sph; however, in a recently published sphingolipidome reference map, analyses of blood plasma, but not lymph, were included (37, 73).

Beyond S1P and TAG concentrations, there remains a persistent paucity of data regarding lymph lipid composition. This incomplete characterization of the two fluids transported by interconnected circulatory systems led us to determine concentrations of SPL in blood plasma and corresponding concentrations in lymph of WT mice. These WT values were then used as a baseline for comparison of SPL detected in blood and lymph of mice lacking expression of the S1P chaperone ApoM (KO) or expressing a human *APOM* transgene (Tg) to determine the possible influence of ApoM-bound S1P on the circulating sphingolipidome. Another reason for this endeavor was our previously reported observation that ApoM KO affected blood S1P concentrations but had no effect on lymph S1P (7), which we confirmed in the studies reported herein (**Figure 4**). Although there was a trend of increased blood Sph in KO and decreased blood Sph in Tg, inversely correlating to blood S1P concentrations, it is still unclear why S1P does not change in KO or Tg lymph as it does in blood. Blood S1P is produced and secreted primarily from erythrocytes and activated platelets via the transporter Mfsd2b, whereas the majority of lymph S1P is produced by lymphatic endothelium and secreted through SPNS2 (74, 75). In the absence of both ApoM and albumin another apolipoprotein, ApoA4, was found to be the most likely blood S1P chaperone; however, lymph was not examined (2). Since ApoM does not appear to be an S1P chaperone in lymph, future studies must characterize the role of ApoM in lymph and determine whether albumin is the sole carrier of lymph S1P or a yet-to-be-determined protein. That KO or Tg expression of ApoM led to more than twice as many significant differences in lymph than in blood (33 versus 14 SPL species significantly different in KO or Tg compared to WT) further emphasizes the need to include lymph in efforts to deploy sphingolipidomics for characterization of pathologies or biomarker identification. This also implies that the changes are not due to intrinsic effects on circulating blood cell utilization of SPL but are more likely to have resulted from altered SPL metabolism by lymphatic versus vascular endothelium, although detailed studies of ApoM effects on SPL metabolism in specific tissues are required.

In addition to S1P and Sph, our characterization of WT blood and lymph SPL included species conventionally considered the direct precursor molecules of S1P (dhSph, dhCer, Cer, and SM) as well as lipids with the potential to alter flux along the biosynthetic pathway as products and/or precursors, and structurally similar lipids known to bind to S1P chaperones or activate S1P receptors (**Figure 1**; dhS1P, dhSM, αOHCer, and C1P). The dramatic impact of ApoM expression on immune development, specifically lymphocyte progenitor proliferation, and cardiovascular biology led us to anticipate more significant differences in measurements from KO or Tg animals. Tight regulation of SPL metabolic flux combined with constitutive deletion or overexpression of ApoM likely resulted in activation of compensatory mechanisms to maintain homeostasis. A model of inducible ApoM knockout or overexpression could be capable of triggering greater perturbations in the SPL metabolic pathways.

The most surprising data obtained from our analyses were the presence of the αOHCer species in blood plasma and their high abundance in lymph plasma. Hydroxylated Cer, such as αOHCer, are considered uncommon and high concentrations are restricted to specific tissues, such as brain, skin, gut, and kidney (76–78). In some cell types, αOHCer and other αOH SPL may regulate cell cycle and apoptotic responses, since lower concentrations of αOHCer are required for induction of apoptosis *in vitro* compared to non-hydroxylated Cer (79, 80). The canonical pathway for αOHCer synthesis is fatty acid alpha-hydroxylase (FA2H) generation of an α-hydroxylated fatty acid from which the αOH fatty acyl-CoA is produced, but studies utilizing knockout animals and samples from patients with *FA2H* mutations have demonstrated that FA2H is required for αOH SPL synthesis in some organs but not others (31, 81). αOHCer serve as precursors for the αOH glycosphingolipids required for myelin sheath maintenance and mutations in *FA2H* have been identified in patients with neurological disease and correlate with clinical signs of demyelination (31, 82). However, while fibroblasts from patients with *FA2H* mutations produced less than half the αOH-SM of control fibroblasts, only a single αOH-SM species was decreased in patient erythrocytes and lymphocyte αOH-SM production was indistinguishable regardless of *FA2H* mutational status, indicating there must be another enzyme responsible for SPL alpha-hydroxylation in blood cells (83). Additionally, whereas upregulation of FA2H was necessary for differentiation of human keratinocytes *in vitro*, studies of *Fa2h* LacZ reporter mice showed that sebocytes were the primary expressors of *Fa2h* and not keratinocytes, and total αOH SPL were unchanged in the skin of *Fa2h* knockout mice (84, 85). While data indicate that FA2H cannot be the sole generator of αOHCer precursor molecules, the identities of the other α-hydroxylating enzymes involved in αOH SPL synthesis are unknown, as potential candidates are believed to have substrate restrictions and would require additional precursor and/or product trafficking steps due to their subcellular localization, e.g. phytanoyl-CoA 2-hydroxylase expression is restricted to peroxisomes and metabolizes branched acyl CoAs; stearoyl-CoA desaturase-1 introduces *cis*-bonds at the C9 rather than the C2 position (86, 87). This class of SPL was also the most affected by ApoM KO or Tg, particularly in the lymph. To our knowledge, this is the first report of αOHCer detection in blood or lymph. Thus, the source of αOHCer in either compartment and the import of circulating αOHCer concentration dynamics are yet to be explored.

There are some limitations to be considered for this report. Although SMs are the most abundant SPL in HDL, SM concentrations decrease as HDL particle density increases, whereas S1P concentrations are higher in smaller (usually denser) HDL particles (88). DAG can be produced as a direct consequence of SM synthesis from Cer and can subsequently be acylated to form TAG, but circulating DAGs and TAGs are likely primarily obtained from dietary sources (89, 90). Circulating TAGs, particularly in lymph, largely reflect dietary intake and their concentrations in unfasted animals vary widely (91, 92). While TAG metabolism and cholesterol efflux pathways have been linked to ApoM blood concentrations (43, 44, 93), a primary goal of this study was to provide the first characterization and comparison of SPL in blood versus lymph plasma. Thus, since TAG was another step removed from SPL metabolism and measurements are confounded by diet and fasting, we did not include their measurement in this study.

Other SPL and lipids that we did not address in this study were the glycosphingolipids (GSL) or glycerolipids. GSL are formed from Cer or αOHCer by covalent linkage of one or more sugar moieties to the Cer backbone, beginning with either glucose or galactose (94, 95). GSL are the most diverse group of glycolipids and serve to modify cell and organelle membranes, interacting with cholesterol, phospholipids, glycerophospholipids, and other SPL to alter membrane density and form membrane domains for signaling platforms (94). Defects in GSL synthesis can result in rare genetic diseases, such as those seen in patients mentioned above unable to produce αOH GSL, leading to ataxia and myelin degradation (82, 83). Conversely, lysosomal storage diseases such as Tay-Sachs, Sandhoff, Krabbe, and Fabry disease result from defective GSL catabolism and may also affect the integumentary, nervous, renal, and digestive systems (29, 95, 96). Alterations in the metabolism of Cer to SM can also result in the accumulation of GSL. Knockout of the genes for SMS1 or SMS2 illustrated their differential contribution to the balance of circulating SPL concentrations: plasma from *Sms1^-/-^* (which associates with glucosylceramide synthase (*Ugcg*)) animals had significantly increased concentrations of GSL but unchanged Cer, whereas *Sms2^-/-^* animals had significantly increased Cer and wildtype levels of GSL (97). Future studies of animals with altered ApoM expression should determine if the changes we identified in blood or particularly in lymph result in altered GSL, thus contributing to the previously reported autoimmune, vascular, or metabolic phenotypes (7, 8, 44).

Lastly, collecting mouse lymph is not trivial: upon finding the cisterna chyli, if lymph is collected uncontaminated by blood, the average volume obtained is less than 2 µl. Lipidomic technologies are not yet capable of interrogating such a small volume, necessitating pooling of samples. While the low N is not optimal, we believe these results provide increased awareness of the true complexity of lymph and blood plasmas and a starting point for researchers in diverse fields to investigate previously overlooked SPL species.

## Data Availability

The original contributions presented in the study are included in the article/**Supplementary Material**. Further inquiries can be directed to the corresponding author.
