# Supplementary material for: Apolipoprotein M expression modifies the sphingolipid landscape in murine blood and lymph
Source: Front Immunol. 2025 May 2;16:1572959. doi: 10.3389/fimmu.2025.1572959 (PMC12081406; doi:10.3389/fimmu.2025.1572959)
Supplement: Supplementary file 1 [file Table1.docx]

| **Supplemental Table 2: Sphingoid base (SB) species concentrations in blood and lymph** | | | | | | | | | | | | |
| --- | --- | --- | --- | --- | --- | --- | --- | --- | --- | --- | --- | --- |
| Values (nM) for individual pooled samples ("A" and "B") are averaged to yield the mean and standard deviation (SD) for each species.  WT = wild-type, KO = *Apom^-/-^*, Tg = *APOM* transgenic mice. ***** Mean is statistically different from WT mean based on unpaired *t*-test. a: Value is from a sample whose signal was one to two times that of the matrix blank. b: Value is from a sample whose signal was below that of the matrix blank. It is excluded from further statistical analysis. c: Value is from a sample whose signal was below the detection limit. It is excluded from further statistical analysis. | | | | | | | | | | | | |
|  |  | **WT** | | | **KO** | | | | **Tg** | | | |
|  | species (nM) | **mean (SD)** | A | B | **mean (SD)** | A | B | *p*-value | **mean (SD)** | A | B | *p*-value |
| blood | S1P | **552.63 (235.93)** | 719.46 | 385.80 | ***153.93 (17.58)** | 141.50 | 166.37 | 0.0021 | ***1098.18 (79.29)** | 1154.25 | 1042.11 | 0.0005 |
|  | dhS1P | **151.14 (83.19)** | 209.96 | 92.31 | **74.81 (5.13)** | 71.18 | 78.43 |  | **334.24 (35.56)** | 309.09 | 359.38 |  |
|  | Sph | **139.03 (14.78)** | 128.57 | 149.48 | **244.47 (10.93)** | 236.74 | 252.20 |  | **71.51 (48.14)** | 105.56 | 37.47 |  |
|  | dhSph | **74.62 (7.95)** | 80.24 | 69.00 | **96.90 (0.51)** | 97.26 | 96.54 |  | **42.72 (25.50)** | 60.75 | 24.69 |  |
|  |  |  |  |  |  |  |  |  |  |  |  |  |
| lymph | S1P | **52.13 (10.81)** | 59.77 | 44.48 | **66.58 (17.00)** | 54.56 | 78.60 |  | **116.48 (37.19)** | 90.18 | 142.77 |  |
|  | dhS1P | **––** | 31.81^b^ | c | **––** | 9.53^b^ | 20.44^b^ |  | **––** | 8.30^b^ | c |  |
|  | Sph | **265.35 (44.27)** | 296.66 | 234.05^a^ | **319.88 (169.16)** | 200.27^a^ | 439.50 |  | **267.09 (108.48)** | 190.38^a^ | 343.79 |  |
|  | dhSph | **288.20 (30.85)** | 310.01^a^ | 266.38^a^ | **265.95 (47.04)** | 232.69^a^ | 299.21^a^ |  | **197.69** | 115.08^b^ | 197.69^a^ |  |

| **Supplemental Table 3: Ceramide (Cer) species concentrations in blood and lymph** | | | | | | | | | | | | |
| --- | --- | --- | --- | --- | --- | --- | --- | --- | --- | --- | --- | --- |
| Values (nM) for individual pooled samples ("A" and "B") are averaged to yield the mean and standard deviation (SD) for each species.  WT = wild-type, KO = *Apom^-/-^*, Tg = *APOM* transgenic mice. ***** Mean is statistically different from WT mean based on unpaired *t*-test. a: Value is from a sample whose signal was one to two times that of the matrix blank. b: Value is from a sample whose signal was below that of the matrix blank. It is excluded from further statistical analysis. c: Value is from a sample whose signal was below the detection limit. It is excluded from further statistical analysis. | | | | | | | | | | | | |
|  |  | **WT** | | | **KO** | | | | **Tg** | | | |
|  | species (nM) | **mean (SD)** | A | B | **mean (SD)** | A | B | *p*-value | **mean (SD)** | A | B | *p*-value |
| ­­­blood | C14 | **4.49 (1.91)** | 5.84 | 3.14^a^ | **3.42 (0.26)** | 3.60^a^ | 3.24^a^ |  | **4.37** | 4.37 | 1.18^b^ |  |
|  | C16 | **46.15 (12.61)** | 55.07 | 37.24 | **56.00 (3.79)** | 53.32 | 58.68 |  | **44.73 (1.90)** | 43.39 | 46.08 |  |
|  | C18 | **14.97 (3.12)** | 12.76 | 17.17 | **13.44 (2.01)** | 12.02 | 14.86 |  | **22.58 (6.75)** | 17.81 | 27.36 |  |
|  | C18:1 | **3.16 (0.17)** | 3.28 | 3.05 | **5.31 (1.39)** | 6.29 | 4.33 |  | **3.28 (0.34)** | 3.52 | 3.04 |  |
|  | C20 | **22.44 (4.82)** | 25.85 | 19.03 | **18.78 (2.83)** | 20.77 | 16.78 |  | **27.51 (4.23)** | 24.52 | 30.50 |  |
|  | C20:1 | **2.66 (1.88)** | 3.98 | 1.33 | **2.80 (0.30)** | 3.02 | 2.59 |  | **2.40 (1.74)** | 1.16 | 3.63 |  |
|  | C20:4 | **––** | c | c | **––** | c | 0.40^b^ |  | **––** | 0.16^b^ | c |  |
|  | C22 | **128.39 (44.45)** | 159.82 | 96.96 | **121.71 (27.81)** | 141.37 | 102.04 |  | **122.02 (23.52)** | 105.39 | 138.65 |  |
|  | C22:1 | **17.91 (5.80)** | 22.01 | 13.81 | **15.77 (5.52)** | 19.67 | 11.87 |  | **12.41 (0.33)** | 12.17 | 12.64 |  |
|  | C24 | **50.45 (16.05)** | 61.80 | 39.11 | **48.80 (12.25)** | 57.46 | 40.13 |  | **51.15 (5.92)** | 46.97 | 55.34 |  |
|  | C24:1 | **340.50 (63.62)** | 385.48 | 295.52 | **350.12 (12.93)** | 359.27 | 340.98 |  | ***407.91 (53.15)** | 370.32 | 445.49 | 0.0037 |
|  | C26 | **1.60 (0.93)** | 2.26 | 0.94^a^ | **2.69 (0.30)** | 2.90 | 2.48 |  | **2.75 (1.88)** | 1.42^a^ | 4.08 |  |
|  | C26:1 | **2.66 (0.55)** | 2.27 | 3.05 | **2.63 (0.68)** | 3.11 | 2.15 |  | **3.26 (0.28)** | 3.06 | 3.46 |  |
|  |  |  |  |  |  |  |  |  |  |  |  |  |
| ­lymph | C14 | **152.85 (9.06)** | 146.44 | 159.25 | **263.00 (166.52)** | 145.25^a^ | 380.75 |  | **145.94 (41.01)** | 116.94 | 174.94 |  |
|  | C16 | **2222.92 (636.60)** | 1772.77 | 2673.06 | ***1707.43 (357.22)** | 1454.84 | 1960.03 | 0.0222 | ***1328.63 (234.27)** | 1494.29 | 1162.98 | <0.0001 |
|  | C18 | **199.37 (39.24)** | 171.63^a^ | 227.12 | **463.11** | 109.47^b^ | 463.11 |  | **134.09 (30.70)** | 155.79^a^ | 112.38^a^ |  |
|  | C18:1 | **79.09 (3.50)** | 81.57 | 76.61 | **204.69 (222.01)** | 47.71 | 361.68 |  | **28.71 (6.32)** | 24.24 | 33.18 |  |
|  | C20 | **150.81 (26.65)** | 169.66^a^ | 131.97^a^ | **445.34 (433.80)** | 138.59^a^ | 752.08 |  | **––** | 63.86^b^ | 53.45^b^ |  |
|  | C20:1 | **53.22 (16.40)** | 41.63 | 64.82 | **81.62 (59.46)** | 39.58 | 123.67 |  | **66.53 (24.86)** | 84.11 | 48.95 |  |
|  | C20:4 | **––** | c | c | **––** | c | c |  | **––** | c | c |  |
|  | C22 | **485.44 (89.64)** | 422.05 | 548.83 | **398.26 (193.79)** | 261.23 | 535.29 |  | **312.10 (60.26)** | 354.72 | 269.49 |  |
|  | C22:1 | **206.91 (14.62)** | 217.25 | 196.57 | **187.92 (78.02)** | 132.74 | 243.09 |  | **130.76 (48.65)** | 165.16 | 96.36 |  |
|  | C24 | **603.21 (107.04)** | 678.90 | 527.51 | **386.48 (168.38)** | 267.42 | 505.55 |  | **355.23 (108.55)** | 431.99 | 278.48 |  |
|  | C24:1 | **325.94 (0.46)** | 325.62 | 326.27 | **246.43 (132.37)** | 152.83 | 340.03 |  | **246.13 (31.73)** | 223.69 | 268.57 |  |
|  | C26 | **––** | 5.96^b^ | 9.59^b^ | **54.00** | 21.47^b^ | 54.00^a^ |  | **––** | 12.89^b^ | 7.79^b^ |  |
|  | C26:1 | **7.19 (1.23)** | 8.06^a^ | 6.32^a^ | **60.82 (33.94)** | 36.83 | 84.82 |  | **10.20 (6.51)** | 5.60^a^ | 14.81 |  |

| **Supplemental Table 4: Dihydroceramide (dhCer) species concentrations in blood and lymph** | | | | | | | | | | | | |
| --- | --- | --- | --- | --- | --- | --- | --- | --- | --- | --- | --- | --- |
| Values (nM) for individual pooled samples ("A" and "B") are averaged to yield the mean and standard deviation (SD) for each species.  WT = wild-type, KO = *Apom^-/-^*, Tg = *APOM* transgenic mice. ***** Mean is statistically different from WT mean based on unpaired *t*-test. a: Value is from a sample whose signal was one to two times that of the matrix blank. b: Value is from a sample whose signal was below that of the matrix blank. It is excluded from further statistical analysis. c: Value is from a sample whose signal was below the detection limit. It is excluded from further statistical analysis. | | | | | | | | | | | | |
|  |  | **WT** | | | **KO** | | | | **Tg** | | | |
|  | species (nM) | **mean (SD)** | A | B | **mean (SD)** | A | B | *p*-value | **mean (SD)** | A | B | *p*-value |
| blood | C12 | **0.39** | 0.04^b^ | 0.39 | **0.11** | 0.11^a^ | 0.07^b^ |  | **0.81 (0.63)** | 0.37 | 1.26 |  |
|  | C14 | **2.08 (0.73)** | 2.59 | 1.56 | **2.85 (1.54)** | 1.76 | 3.94 |  | **4.10 (4.63)** | 7.38 | 0.82 |  |
|  | C16 | **43.69 (21.81)** | 59.11 | 28.27 | **37.27 (1.05)** | 36.53 | 38.02 |  | **28.11 (12.69)** | 37.09 | 19.14 |  |
|  | C18 | **7.64 (3.54)** | 10.15 | 5.14^a^ | **12.95 (4.50)** | 9.77 | 16.13 |  | **11.90 (1.98)** | 10.49 | 13.30 |  |
|  | C18:1 | **1.59 (1.01)** | 2.30 | 0.87^a^ | **3.76 (3.39)** | 1.36 | 6.15 |  | **1.81 (0.99)** | 2.51 | 1.11 |  |
|  | C20 | **26.80 (18.87)** | 40.14 | 13.46 | **29.06 (4.48)** | 32.23 | 25.89 |  | **46.75 (1.24)** | 47.62 | 45.88 |  |
|  | C20:1 | **3.05 (0.10)** | 3.12 | 2.97 | **7.60 (1.55)** | 6.50 | 8.70 |  | **10.82 (1.08)** | 10.06 | 11.58 |  |
|  | C22 | **35.72 (24.34)** | 52.94 | 18.51 | **43.48 (4.87)** | 40.04 | 46.92 |  | **41.94 (4.24)** | 44.93 | 38.94 |  |
|  | C22:1 | **155.41 (57.54)** | 196.10 | 114.73 | **146.83 (35.99)** | 172.27 | 121.38 |  | **147.20 (30.48)** | 125.65 | 168.76 |  |
|  | C24 | **101.20 (56.19** | 140.93 | 61.47 | **124.10 (7.47)** | 118.82 | 129.39 |  | **105.99 (15.64)** | 117.05 | 94.93 |  |
|  | C24:1 | **404.97 (104.58)** | 478.92 | 331.02 | **393.38 (98.80)** | 463.25 | 323.52 |  | **391.29 (31.64)** | 368.91 | 413.66 |  |
|  | C26 | **22.14** | 22.14 | 4.81^b^ | **16.14 (6.68)** | 11.41^a^ | 20.86 |  | **21.26 (0.48)** | 20.92 | 21.59 |  |
|  | C26:1 | **5.80** | 5.80^a^ | 3.15^b^ | **6.19 (1.55)** | 5.10^a^ | 7.29 |  | **8.54 (1.69)** | 9.74 | 7.34 |  |
|  |  |  |  |  |  |  |  |  |  |  |  |  |
| lymph | C12 | **4.87** | 0.86^b^ | 4.87^a^ | **31.30 (38.42)** | 58.46 | 4.13^a^ |  | **––** | 0.77^b^ | 1.89^b^ |  |
|  | C14 | **61.40 (2.81)** | 59.41 | 63.39 | **61.19** | 2.60^b^ | 61.19 |  | **19.36 (12.30)** | 28.06 | 10.66 |  |
|  | C16 | **1938.79 (85.96)** | 1878.01 | 1999.58 | ***1038.70 (631.29)** | 592.30 | 1485.09 | 0.0481 | ***1099.94 (340.74)** | 1340.88 | 859.00 | 0.0003 |
|  | C18 | **192.93 (62.84)** | 237.36 | 148.49^a^ | **483.42** | 112.69^b^ | 438.42 |  | **92.79** | 92.79^a^ | 72.77^b^ |  |
|  | C18:1 | **28.83 (14.35)** | 18.69^a^ | 38.98 | **38.04 (15.68)** | 26.96^a^ | 49.13 |  | **––** | 2.27^b^ | 2.56^b^ |  |
|  | C20 | **909.28 (27.57)** | 889.78 | 928.77 | **1509.99 (1470.73)** | 470.03 | 2549.96 |  | ***420.15 (25.96)** | 438.51 | 401.80 | 0.0183 |
|  | C20:1 | **157.76 (58.97)** | 116.06 | 199.46 | **205.91 (15.06)** | 216.56 | 195.26 |  | **114.38 (48.21)** | 148.47 | 80.30 |  |
|  | C22 | **1342.29 (158.96)** | 1454.69 | 1229.89 | **936.15 (322.86)** | 707.86 | 1164.44 |  | ***702.98 (236.28)** | 870.05 | 535.90 | 0.0032 |
|  | C22:1 | **445.16 (82.21)** | 387.02 | 503.29 | **365.21 (177.71)** | 239.55 | 490.87 |  | **287.28 (53.75)** | 325.28 | 249.27 |  |
|  | C24 | **2371.41 (380.67)** | 2640.58 | 2102.24 | **1802.23 (702.16)** | 1305.73 | 2298.73 |  | ***1268.12 (515.66)** | 1632.75 | 903.49 | <0.0001 |
|  | C24:1 | **477.03 (29.05)** | 456.49 | 497.57 | **437.27 (247.94)** | 261.95 | 612.59 |  | **307.09 (44.93)** | 338.86 | 275.32 |  |
|  | C26 | **261.35** | 261.35^a^ | 154.69^b^ | **675.57** | 168.08^b^ | 675.57 |  | **198.45** | 166.97^b^ | 198.45^a^ |  |
|  | C26:1 | **––** | 18.04^b^ | 14.67^b^ | **253.48** | 20.14^b^ | 253.48^a^ |  | **––** | 21.75^b^ | 24.41^b^ |  |

| **Supplemental Table 5: Alpha-hydroxyceramide (αOHCer) species concentrations in blood and lymph** | | | | | | | | | | | | |
| --- | --- | --- | --- | --- | --- | --- | --- | --- | --- | --- | --- | --- |
| Values (nM) for individual pooled samples ("A" and "B") are averaged to yield the mean and standard deviation (SD) for each species.  WT = wild-type, KO = *Apom^-/-^*, Tg = *APOM* transgenic mice. ***** Mean is statistically different from WT mean based on unpaired *t*-test. a: Value is from a sample whose signal was one to two times that of the matrix blank. b: Value is from a sample whose signal was below that of the matrix blank. It is excluded from further statistical analysis. c: Value is from a sample whose signal was below the detection limit. It is excluded from further statistical analysis. | | | | | | | | | | | | |
|  |  | **WT** | | | **KO** | | | | **Tg** | | | |
|  | species (nM) | **mean (SD)** | A | B | **mean (SD)** | A | B | *p*-value | **mean (SD)** | A | B | *p*-value |
| blood | C14 | **62.41 (14.35)** | 52.27 | 72.56 | **77.95 (5.08)** | 74.36 | 81.54 |  | **77.42 (8.18)** | 83.20 | 71.64 |  |
|  | C16 | **115.69 (10.77)** | 108.08 | 123.31 | **121.64 (17.50)** | 109.26 | 134.02 |  | **113.44 (28.85)** | 93.04 | 133.84 |  |
|  | C18 | **19.95 (0.92)** | 19.30 | 20.61 | **36.01 (15.27)** | 46.81 | 25.22 |  | **25.06 (9.11)** | 18.62 | 31.51 |  |
|  | C18:1 | **9.01** | 3.03^b^ | 9.01 | **12.83 (4.74)** | 16.18 | 9.47 |  | **8.34** | 2.14^b^ | 8.34 |  |
|  | C20 | **3.05 (0.65)** | 3.51^a^ | 2.59^a^ | **4.86** | 4.86 | 2.10^b^ |  | **4.15 (0.67)** | 3.68^a^ | 4.63 |  |
|  | C20:1 | **––** | 2.53^b^ | 0.77^b^ | **4.51 (0.72)** | 3.99^a^ | 5.02^a^ |  | **––** | 1.82^b^ | 2.16^b^ |  |
|  | C22 | **27.17 (1.01)** | 26.45 | 27.88 | **37.73 (5.25)** | 34.02 | 41.43 |  | **32.16 (8.58)** | 26.09 | 38.23 |  |
|  | C22:1 | **162.13 (12.53)** | 153.28 | 170.99 | ***224.68 (29.63)** | 203.72 | 245.63 | <0.0001 | ***220.99 (71.54)** | 170.40 | 271.57 | 0.0070 |
|  | C24 | **6.35 (4.17)** | 9.30 | 3.39^a^ | **3.31 (0.26)** | 3.49^a^ | 3.12^a^ |  | **11.58 (5.56)** | 15.51 | 7.65 |  |
|  | C24:1 | **43.44 (2.47)** | 45.19 | 41.69 | **44.28 (5.02)** | 40.73 | 47.84 |  | **40.28 (3.48)** | 42.74 | 37.83 |  |
|  | C26 | **––** | 0.19^b^ | c | **––** | 0.33^b^ | 0.11^b^ |  | **1.45** | 0.32^b^ | 1.45^a^ |  |
|  | C26:1 | **5.39 (0.20)** | 5.54 | 5.25 | **4.08 (1.13)** | 3.28 | 4.88 |  | **8.05 (0.51)** | 7.70 | 8.41 |  |
|  |  |  |  |  |  |  |  |  |  |  |  |  |
| lymph | C14 | **2928.87 (84.36)** | 2869.22 | 2988.53 | **2919.57 (61.88)** | 2875.82 | 2963.33 |  | ***2065.92 (28.88)** | 2045.50 | 2086.34 | 0.0003 |
|  | C16 | **8752.78 (409.48)** | 8463.23 | 9042.33 | ***10990.32 (374.58)** | 11255.19 | 10725.45 | <0.0001 | ***6711.76 (60.63)** | 6754.63 | 6668.89 | <0.0001 |
|  | C18 | **793.02 (124.43)** | 881.00 | 705.03 | **553.85 (190.65)** | 688.66 | 419.05 |  | ***282.40 (33.12)** | 258.98 | 305.82 | 0.0180 |
|  | C18:1 | **1641.30 (88.51)** | 1578.72 | 1703.88 | ***981.30 (467.60)** | 1311.94 | 650.66 | 0.0480 | ***797.21 (24.31)** | 814.40 | 780.02 | 0.0003 |
|  | C20 | **2322.08 (406.29)** | 2034.79 | 2609.37 | ***1442.85 (580.42)** | 1853.28 | 1032.43 | 0.0105 | ***1332.77 (392.29)** | 1055.38 | 1610.16 | 0.0001 |
|  | C20:1 | **1314.21 (251.76)** | 1136.19 | 1492.24 | **672.07 (78.03)** | 727.25 | 616.90 |  | ***685.55 (104.14)** | 611.92 | 759.19 | 0.0046 |
|  | C22 | **1906.18 (17.28)** | 1893.96 | 1918.40 | ***869.40 (37.50)** | 895.92 | 842.89 | 0.0032 | ***1282.83 (105.67)** | 1357.55 | 1208.11 | 0.0049 |
|  | C22:1 | **5734.38 (381.47)** | 5464.64 | 6004.13 | ***6925.73 (859.80)** | 6317.76 | 7533.70 | 0.0001 | ***3350.64 (21.81)** | 3335.21 | 3366.06 | <0.0001 |
|  | C24 | **1535.96 (369.42)** | 1797.18 | 1274.74 | ***553.14 (197.75)** | 413.31 | 692.97 | 0.0049 | ***1094.83 (204.12)** | 1239.17 | 950.49 | 0.0382 |
|  | C24:1 | **278.84 (17.15)** | 266.71 | 290.96 | **647.28 (381.86)** | 377.27 | 917.30 |  | **212.84 (46.73)** | 245.88 | 179.79^a^ |  |
|  | C26 | **460.26 (173.97)** | 583.28 | 337.25 | **77.55 (0.43)** | 77.86 | 77.25 |  | **307.69 (35.63)** | 332.88 | 282.49 |  |
|  | C26:1 | **275.30 (21.64)** | 290.60 | 259.99 | **66.37 (22.01)** | 81.95 | 50.81 |  | **112.66 (53.80)** | 150.70 | 74.62 |  |

| **Supplemental Table 6: Ceramide 1-phosphate (C1P) species concentrations in blood and lymph** | | | | | | | | | | | | |
| --- | --- | --- | --- | --- | --- | --- | --- | --- | --- | --- | --- | --- |
| Values (nM) for individual pooled samples ("A" and "B") are averaged to yield the mean and standard deviation (SD) for each species.  WT = wild-type, KO = *Apom^-/-^*, Tg = *APOM* transgenic mice. ***** Mean is statistically different from WT mean based on unpaired *t*-test. a: Value is from a sample whose signal was one to two times that of the matrix blank. b: Value is from a sample whose signal was below that of the matrix blank. It is excluded from further statistical analysis. c: Value is from a sample whose signal was below the detection limit. It is excluded from further statistical analysis. | | | | | | | | | | | | |
|  |  | **WT** | | | **KO** | | | | **Tg** | | | |
|  | **species (nM)** | **mean (SD)** | A | B | **mean (SD)** | A | B | *p*-value | **mean (SD)** | A | B | *p*-value |
| blood | C14 | **6.14 (1.15)** | 6.95 | 5.32 | **6.49 (2.69)** | 8.40 | 4.59 |  | **7.77 (4.27)** | 4.75 | 10.80 |  |
|  | C16 | **626.15 (15.73)** | 637.28 | 615.03 | ***740.49 (83.95)** | 799.85 | 681.13 | 0.0001 | **679.37 (191.90)** | 543.67 | 815.06 |  |
|  | C18 | **208.14 (50.70)** | 243.99 | 172.28 | **243.29 (4.32)** | 246.35 | 240.24 |  | **226.94 (39.25)** | 199.19 | 254.69 |  |
|  | C18:1 | **35.83 (0.08)** | 35.89 | 35.78 | **45.20 (3.90)** | 42.44 | 47.96 |  | **44.17 (4.13)** | 41.25 | 47.08 |  |
|  | C20 | **13.59 (6.67)** | 18.31 | 8.88 | **23.73 (15.33)** | 34.57 | 12.88 |  | **11.66 (4.13)** | 8.74 | 14.58 |  |
|  | C20:1 | **––** | c | c | **––** | c | c |  | **––** | c | c |  |
|  | C22 | **1.07 (0.15)** | 0.96 | 1.18 | **2.15 (0.12)** | 2.07 | 2.23 |  | **1.05 (0.44)** | 1.36 | 0.74 |  |
|  | C22:1 | **2.75 (0.02)** | 2.73 | 2.76 | **3.57** | 3.57 | c |  | **0.52 (0.17)** | 0.39 | 0.64 |  |
|  | C24 | **3.55 (0.06)** | 3.59 | 3.50 | **2.72 (1.40)** | 3.71 | 1.73 |  | **3.42 (1.58)** | 2.31 | 4.54 |  |
|  | C24:1 | **1.72 (0.02)** | 1.73^a^ | 1.71^a^ | **3.56 (3.43)** | 1.14^a^ | 5.99 |  | **1.21 (0.32)** | 0.98^a^ | 1.43^a^ |  |
|  | C26 | **16.00 (0.41)** | 16.29 | 15.71 | **17.32 (0.72)** | 17.82 | 16.81 |  | **17.28 (0.31)** | 17.06 | 17.50 |  |
|  | C26:1 | **6.35** | 6.35^a^ | 3.75^b^ | **5.43** | 3.37^b^ | 5.43^a^ |  | **7.93 (4.48)** | 4.76^a^ | 11.10 |  |
|  |  |  |  |  |  |  |  |  |  |  |  |  |
| lymph | C14 | **250.96 (18.42)** | 237.94 | 263.99 | **403.18 (256.77)** | 221.62 | 584.75 |  | **191.79 (29.30)** | 171.08 | 212.51 |  |
|  | C16 | **4834.36 (583.68)** | 4421.64 | 5247.09 | **5305.02 (1652.66)** | 4136.41 | 6473.63 |  | **4955.95 (1293.07)** | 5870.29 | 4041.61 |  |
|  | C18 | **837.16 (20.03)** | 823.00 | 851.32 | **1639.64 (1651.77)** | 471.66 | 2807.62 |  | **869.64 (266.05)** | 1057.77 | 681.51 |  |
|  | C18:1 | **93.73 (66.82)** | 46.48^a^ | 140.97 | **268.87 (289.18)** | 64.39^a^ | 473.35 |  | **96.58 (63.63)** | 141.57 | 51.59 |  |
|  | C20 | **81.42** | 81.42^a^ | 55.42^b^ | **326.58 (294.92)** | 118.04^a^ | 535.11 |  | **95.04 (31.14)** | 73.02^a^ | 117.06^a^ |  |
|  | C20:1 | **3.74** | c | 3.74 | **––** | c | c |  | **9.84 (12.01)** | 18.33 | 1.35^a^ |  |
|  | C22 | **48.69 (32.78)** | 71.87 | 25.52 | **123.29 (117.31)** | 40.34 | 206.24 |  | **49.37 (49.66)** | 84.49 | 14.26^a^ |  |
|  | C22:1 | **––** | c | c | **39.47 (48.71)** | 5.02^a^ | 73.91 |  | **9.86 (1.91)** | 11.21 | 8.51 |  |
|  | C24 | **90.39 (1.84)** | 89.09 | 91.69 | **230.36 (143.29)** | 129.04 | 331.68 |  | **115.29 (1.00)** | 114.58 | 115.99 |  |
|  | C24:1 | **39.44** | 39.44^a^ | 9.33^b^ | **76.81** | 29.05^b^ | 76.81 |  | **44.50** | 44.50^a^ | 18.35^b^ |  |
|  | C26 | **423.07 (46.31)** | 455.81 | 390.32 | **782.69 (317.92)** | 557.89 | 1007.49 |  | **321.01 (39.60)** | 349.01 | 293.01 |  |
|  | C26:1 | **248.33** | 248.33^a^ | 123.81^b^ | **400.17** | 145.75^b^ | 400.17 |  | **159.88 (12.68)** | 168.84^a^ | 150.92^a^ |  |

| **Supplemental Table 7: Diacylglycerol (DAG) species concentrations in blood and lymph** | | | | | | | | | | | | |
| --- | --- | --- | --- | --- | --- | --- | --- | --- | --- | --- | --- | --- |
| Values (nM) for individual pooled samples ("A" and "B") are averaged to yield the mean and standard deviation (SD) for each species.  WT = wild-type, KO = *Apom^-/-^*, Tg = *APOM* transgenic mice. ***** Mean is statistically different from WT mean based on unpaired *t*-test. a: Value is from a sample whose signal was one to two times that of the matrix blank. b: Value is from a sample whose signal was below that of the matrix blank. It is excluded from further statistical analysis. c: Value is from a sample whose signal was below the detection limit. It is excluded from further statistical analysis. | | | | | | | | | | | | |
|  |  | **WT** | | | **KO** | | | | **Tg** | | | |
|  | **species (nM)** | **mean (SD)** | A | B | **mean (SD)** | A | B | *p*-value | **mean (SD)** | A | B | *p*-value |
| blood | di-C14 | **7.5 (2.5)** | 5.8 | 9.3 | **16.1 (14.3)** | 6.0 | 26.2 |  | **10.4 (3.6)** | 13.0 | 7.8 |  |
|  | C14:0/16:0 | **35.0 (7.4)** | 29.8 | 40.2 | **66.3 (40.5)** | 37.7 | 95.0 |  | **63.3 (1.9)** | 61.9 | 64.6 |  |
|  | C14:0/18:0 | **40.0 (25.6)** | 22.0 | 58.1 | **66.9 (37.4)** | 40.5 | 93.3 |  | **60.8 (5.6)** | 64.7 | 56.8 |  |
|  | C14:0/18:1 | **3.9 (0.3)** | 4.1 | 3.7 | **6.6 (0.5)** | 7.0 | 6.3 |  | **6.9 (5.4)** | 3.0 | 10.7 |  |
|  | di-C16:0 | **75.0 (28.1)** | 55.1 | 94.8 | **138.8 (67.7)** | 90.9 | 186.7 |  | ***133.3 (12.3)** | 124.6 | 142.1 | 0.0168 |
|  | C16:0/18:0 | **195.6 (71.1)** | 145.3 | 245.9 | **331.4 (170.0)** | 211.3 | 451.6 |  | **237.3 (8.2)** | 231.5 | 243.2 |  |
|  | C16:0/18:1 | **80.6 (0.2)** | 80.8 | 80.5 | **128.5 (62.9)** | 172.9 | 84.0 |  | ***181.2 (111.8)** | 102.1 | 260.3 | 0.0001 |
|  | C16:0/24:1 | **0.8 (0.3)** | 1.1 | 0.6 | **0.8 (0.8)** | 1.4 | 0.3 |  | **0.7 (0.8)** | 0.1^a^ | 1.3 |  |
|  | di-C16:1 | **9.2 (0.6)** | 8.8 | 9.7 | **13.7 (2.4)** | 12.0 | 15.4 |  | **12.0 (0.4)** | 11.7 | 12.3 |  |
|  | C16:1/18:0 | **3.8 (1.8)** | 2.5 | 5.0 | **2.8 (0.1)** | 2.9 | 2.8 |  | **5.7 (2.2)** | 4.2 | 7.2 |  |
|  | C16:1/18:1 | **29.2 (1.3)** | 28.2 | 30.1 | **40.4 (17.0)** | 52.4 | 28.4 |  | **35.3 (15.0)** | 24.7 | 45.9 |  |
|  | C16:1/20:0 | **13.53** | 0.7^b^ | 13.5 | **19.5** | 0.7^b^ | 19.5 |  | **10.5 (3.1)** | 8.3 | 12.7 |  |
|  | C16:1/24:1 | **––** | c | 0.1^b^ | **––** | c | c |  | **––** | 0.1^b^ | c |  |
|  | di-C18:0 | **33.0 (13.1)** | 23.7 | 42.3 | **46.7 (20.6)** | 32.2 | 61.3 |  | **42.6 (0.9)** | 43.2 | 42.0 |  |
|  | C18:0/18:1 | **13.2 (2.4)** | 11.5 | 15.0 | **19.2 (3.5)** | 21.6 | 16.7 |  | **20.7 (14.8)** | 10.2 | 31.1 |  |
|  | C18:0/18:2 | **15.5 (0.5)** | 15.9 | 15.2 | **24.6 (6.3)** | 29.1 | 20.1 |  | **20.7 (4.3)** | 17.6 | 23.7 |  |
|  | C18:0/20:4 | **14.0 (5.3)** | 10.3 | 17.8 | **26.6 (7.9)** | 32.1 | 21.0 |  | **14.0 (0.2)** | 13.9 | 14.1 |  |
|  | di-C18:1 | **12.5 (2.8)** | 10.5 | 14.6 | **21.0 (1.6)** | 22.2 | 19.9 |  | **15.6 (5.4)** | 11.7 | 19.4 |  |
|  | C18:1/24:0 | **0.8 (0.4)** | 0.5 | 1.1 | **0.8 (0.04)** | 0.7 | 0.8 |  | **1.7 (1.4)** | 0.8 | 2.7 |  |
|  | C18:1/24:1 | **0.2 (0.1)** | 0.1 | 0.2 | **1.2 (0.4)** | 1.5 | 1.0 |  | **1.3 (1.0)** | 0.6 | 1.9 |  |
|  |  |  |  |  |  |  |  |  |  |  |  |  |
| lymph | di-C14 | **348.4 (72.1)** | 399.3 | 297.4 | **340.0 (74.6)** | 392.8 | 287.2 |  | **233.3 (16.6)** | 245.0 | 221.6 |  |
|  | C14:0/16:0 | **3226.6 (728.5)** | 3741.7 | 2711.5 | **2947.6 (816.6)** | 3525.0 | 2370.1 |  | ***1828.7 (347.2)** | 1583.1 | 2074.2 | 0.0154 |
|  | C14:0/18:0 | **3611.1 (143.9)** | 3712.9 | 3509.3 | **4532.5 (1570.4)** | 5643.0 | 3422.0 |  | ***2173.2 (52.4)** | 2136.2 | 2210.3 | 0.0129 |
|  | C14:0/18:1 | **560.8 (25.9)** | 579.1 | 542.4 | **493.5 (8.1)** | 499.2 | 487.7 |  | **459.7 (62.9)** | 504.1 | 415.2 |  |
|  | di-C16:0 | **9064.9 (822.1)** | 8483.6 | 9646.2 | **9105.9 (1787.3)** | 10369.7 | 7842.1 |  | ***6214.3 (109.8)** | 6291.9 | 6136.6 | <0.0001 |
|  | C16:0/18:0 | **15052.6 (1395.7)** | 16039.5 | 14065.7 | **17263.9 (7634.0)** | 22662.0 | 11865.9 |  | ***10220.8 (681.0)** | 10702.3 | 9739.3 | <0.0001 |
|  | C16:0/18:1 | **8561.0 (1316.1)** | 7630.3 | 9491.6 | **6591.4 (639.2)** | 6139.4 | 7043.4 |  | ***6145.0 (1362.0)** | 7108.1 | 5181.9 | 0.0001 |
|  | C16:0/24:1 | **18.9 (9.4)** | 12.2 | 25.6 | **21.5 ±(3.4)** | 31.0 | 12.1 |  | **24.3 (14.2)** | 34.3 | 14.3 |  |
|  | di-C16:1 | **391.2 (19.0)** | 404.6 | 377.7 | **321.9 (34.0)** | 346.0 | 297.9 |  | **301.0 (23.7)** | 284.2 | 317.7 |  |
|  | C16:1/18:0 | **363.6 (100.3)** | 292.7 | 434.6 | **252.8 (40.3)** | 281.3 | 224.3 |  | **307.0 (127.8)** | 397.4 | 216.6 |  |
|  | C16:1/18:1 | **1096.8 (388.5)** | 822.1 | 1371.6 | **995.4 (43.4)** | 1026.1 | 964.7 |  | **718.8 (158.0)** | 830.5 | 607.1 |  |
|  | C16:1/20:0 | **2179.2 (987.7)** | 1480.8 | 2877.6 | **1873.4 (73.1)** | 1821.7 | 1925.0 |  | **2377.1 (458.8)** | 2701.5 | 2052.7 |  |
|  | C16:1/24:1 | **––** | c | 6.2^b^ | **––** | 5.7^b^ | 5.0^b^ |  | **––** | 0.4^b^ | c |  |
|  | di-C18:0 | **2002.8 (514.5)** | 2366.6 | 1639.0 | **2491.6 (1026.2)** | 3217.3 | 1766.0 |  | **1499.8 (114.9)** | 1418.5 | 1581.1 |  |
|  | C18:0/18:1 | **1720.0 (90.3)** | 1656.1 | 1783.8 | **1156.1 (205.5)** | 1301.4 | 1010.8 |  | **1016.5 (208.8)** | 1164.1 | 868.9 |  |
|  | C18:0/18:2 | **4806.9 (873.3)** | 4189.3 | 5424.4 | **2728.4 (11.1)** | 2736.2 | 2720.5 |  | **3408.0 (1131.0)** | 4207.8 | 2608.3 | 0.0154 |
|  | C18:0/20:4 | **565.9 (103.4)** | 492.8 | 639.0 | **313.7 (70.1)** | 264.1 | 363.3 |  | **439.7 (58.3)** | 480.9 | 398.4 |  |
|  | di-C18:1 | **682.6 (171.9)** | 561.0 | 804.1 | **446.3 (14.7)** | 456.7 | 435.9 |  | **490.9 (156.7)** | 601.7 | 380.1 |  |
|  | C18:1/24:0 | **57.6 (1.7)** | 58.8 | 56.4 | **45.1 (20.7)** | 59.8 | 30.4 |  | **40.0** | 2.9^b^ | 40.0 |  |
|  | C18:1/24:1 | **30.5 (31.9)** | 7.9 | 53.0 | **17.3 (13.6)** | 26.9 | 7.7 |  | **5.3 (1.7)** | 4.1 | 6.5 |  |

| **Supplemental Table 8: Sphingomyelin (SM) species concentrations in blood and lymph** | | | | | | | | | | | | |
| --- | --- | --- | --- | --- | --- | --- | --- | --- | --- | --- | --- | --- |
| Values (nM) for individual pooled samples ("A" and "B") are averaged to yield the mean and standard deviation (SD) for each species.  WT = wild-type, KO = *Apom^-/-^*, Tg = *APOM* transgenic mice. ***** Mean is statistically different from WT mean based on unpaired *t*-test. a: Value is from a sample whose signal was one to two times that of the matrix blank. b: Value is from a sample whose signal was below that of the matrix blank. It is excluded from further statistical analysis. c: Value is from a sample whose signal was below the detection limit. It is excluded from further statistical analysis. | | | | | | | | | | | | |
|  |  | **WT** | | | **KO** | | | | **Tg** | | | |
|  | **species (nM)** | **mean (SD)** | A | B | **mean (SD)** | A | B | *p*-value | **mean (SD)** | A | B | *p*-value |
| blood | C14 | **193.8 (62.9)** | 238.3 | 149.3 | **169.2 (28.4)** | 149.1 | 189.3 |  | **177.8 (20.6)** | 192.4 | 163.2 |  |
|  | C16 | **16708.8 (2865.1)** | 18734.7 | 14682.8 | **16704.9 (361.0)** | 16960.1 | 16449.6 |  | **16116.7 (296.1)** | 16326.1 | 15907.3 |  |
|  | C18 | **2667.4 (803.5)** | 2099.2 | 3235.5 | **2826.1 (1809.1)** | 1546.8 | 4105.3 |  | **1046.7 (426.0)** | 745.5 | 1347.9 |  |
|  | C18:1 | **723.5 (174.4)** | 600.1 | 846.8 | **876.6 (332.5)** | 641.5 | 1111.7 |  | **636.3 (83.2)** | 695.1 | 577.4 |  |
|  | C20 | **435.9 (153.8)** | 327.1 | 544.6 | **443.6 (119.4)** | 359.1 | 528.0 |  | **346.8 (69.4)** | 297.7 | 395.8 |  |
|  | C20:1 | **254.3 (79.5)** | 198.1 | 310.5 | **291.1 (120.0)** | 206.3 | 376.0 |  | **175.2 (10.0)** | 182.2 | 168.2 |  |
|  | C22 | **4746.1 (416.2)** | 4451.8 | 5040.4 | **4738.2 (716.4)** | 4231.6 | 5244.7 |  | ***2784.2 (973.8)** | 2095.7 | 3472.8 | 0.0198 |
|  | C22:1 | **2669.2 (622.9)** | 2228.7 | 3109.7 | **2274.7 (921.4)** | 1623.2 | 2926.3 |  | **1523.2 (125.7)** | 1612.1 | 1434.3 |  |
|  | C24 | **6504.8 (1314.0)** | 5575.6 | 7433.9 | **7100.0 (1868.6)** | 5778.7 | 8421.4 |  | ***4147.8 (234.8)** | 4313.8 | 3981.7 | 0.0063 |
|  | C24:1 | **24992.2 (974.1)** | 25681.0 | 24303.4 | **24101.6 (9249.0)** | 17561.6 | 30641.6 |  | ***16551.3 (814.5)** | 17127.3 | 15975.4 | <0.0001 |
|  | C26 | **0.9 (0.0)** | 0.8 | 0.9 | **0.5 (0.3)** | 0.3 | 0.8 |  | **0.7 (0.1)** | 0.8 | 0.7 |  |
|  | C26:1 | **0.6 (0.4)** | 0.9 | 0.3 | **0.7 (0.5)** | 0.4 | 1.1 |  | **1.9** | c | 1.9 |  |
|  | Lyso | **1.6** | 1.6 | 0.3^b^ | **––** | 0.2^b^ | c |  | **3.0** | 3.0 | c |  |
|  |  |  |  |  |  |  |  |  |  |  |  |  |
| lymph | C14 | **54.9 (5.8)** | 50.8 | 59.0 | **72.0 (0.5)** | 72.4 | 71.7 |  | **29.0 (31.0)** | 7.1 | 50.9 |  |
|  | C16 | **3839.2 (424.7)** | 3538.9 | 4139.5 | ***2480.0 (749.2)** | 1950.3 | 3009.8 | <0.0001 | **3300.4 (143.6)** | 3198.9 | 3402.0 |  |
|  | C18 | **1072.6 (593.8)** | 652.7 | 1492.5 | **1486.8 (14.3)** | 1496.9 | 1476.7 |  | **1758.9 (2111.4)** | 265.9 | 3251.8 |  |
|  | C18:1 | **71.0 (93.5)** | 4.9 | 137.2 | **129.6 (73.4)** | 77.7 | 181.5 |  | **14.9 (2.3)** | 16.6 | 13.3 |  |
|  | C20 | **392.8 (95.9)** | 325.0 | 460.6 | **115.3 (118.7)** | 199.2 | 31.4 |  | **166.5 (56.7)** | 206.6 | 126.5 |  |
|  | C20:1 | **––** | c | 0.9^b^ | **––** | c | c |  | **––** | 6.0^b^ | 6.6^b^ |  |
|  | C22 | **70.8 (37.1)** | 44.5 | 97.0 | **116.0 (122.0)** | 202.3 | 29.7 |  | **288.2 (165.6)** | 405.2 | 171.1 |  |
|  | C22:1 | **15.0 (2.3)** | 13.4 | 16.6 | **25.4 (18.7)** | 38.6 | 12.2 |  | **148.3 (23.4)** | 131.7 | 164.8 |  |
|  | C24 | **167.0 (121.7)** | 81.0 | 253.0 | **82.2 (21.6)** | 66.9 | 97.5 |  | **235.0 (3.8)** | 232.2 | 237.7 |  |
|  | C24:1 | **157.0 (180.1)** | 284.4 | 29.6 | **202.3 (152.8)** | 310.4 | 94.3 |  | **289.5 (160.2)** | 402.7 | 176.2 |  |
|  | C26 | **––** | c | 1.4^b^ | **––** | 0.9^b^ | c |  | **6.7 (5.0)** | 10.2 | 3.1^a^ |  |
|  | C26:1 | **––** | c | c | **––** | 1.5^b^ | c |  | **16.2 (15.6)** | 27.2 | 5.1 |  |
|  | Lyso | **––** | 5.3^b^ | c | **––** | 9.4^b^ | c |  | **––** | c | 4.6^b^ |  |

| **Supplemental Table 9: Dihydrosphingomyelin (dhSM) species concentrations in blood and lymph** | | | | | | | | | | | | |
| --- | --- | --- | --- | --- | --- | --- | --- | --- | --- | --- | --- | --- |
| Values (nM) for individual pooled samples ("A" and "B") are averaged to yield the mean and standard deviation (SD) for each species.  WT = wild-type, KO = *Apom^-/-^*, Tg = *APOM* transgenic mice. ***** Mean is statistically different from WT mean based on unpaired *t*-test. a: Value is from a sample whose signal was one to two times that of the matrix blank. b: Value is from a sample whose signal was below that of the matrix blank. It is excluded from further statistical analysis. c: Value is from a sample whose signal was below the detection limit. It is excluded from further statistical analysis. | | | | | | | | | | | | |
|  |  | **WT** | | | **KO** | | | | **Tg** | | | |
|  | **species (nM)** | **mean (SD)** | A | B | **mean (SD)** | A | B | *p*-value | **mean (SD)** | A | B | *p*-value |
| blood | C14 | **14.5 (2.5)** | 16.2 | 12.7 | **20.4 (1.3)** | 21.3 | 19.5 |  | **19.1 (3.0)** | 21.2 | 17.1 |  |
|  | C16 | **1217.4 (222.8)** | 1375.0 | 1059.9 | **1212.7 (59.2)** | 1254.5 | 1170.8 |  | **1222.7 (121.1)** | 1308.3 | 1137.1 |  |
|  | C18 | **158.2 (12.9)** | 167.3 | 149.0 | **177.7 (15.5)** | 166.7 | 188.7 |  | **154.3 (9.3)** | 147.7 | 160.9 |  |
|  | C18:1 | **1547.8 (201.0)** | 1690.0 | 1405.7 | **1858.3 (206.2)** | 1712.5 | 2004.1 |  | **1752.8 (210.0)** | 1604.3 | 1901.3 |  |
|  | C20 | **104.7 (5.6)** | 108.7 | 100.8 | **95.7 (2.7)** | 93.8 | 97.7 |  | **80.4 (2.0)** | 79.0 | 81.9 |  |
|  | C20:1 | **550.3 (30.5)** | 571.8 | 528.7 | **547.9 (30.7)** | 526.3 | 569.6 |  | **435.5 (17.4)** | 423.3 | 447.8 |  |
|  | C22 | **591.5 (138.0)** | 689.1 | 494.0 | **456.8 (45.4)** | 488.9 | 424.7 |  | **401.7 (0.9)** | 401.1 | 402.3 |  |
|  | C22:1 | **7135.4 (1806.7)** | 8412.9 | 5857.9 | ***5690.5 (873.4)** | 6308.1 | 5072.9 | 0.0264 | ***4746.0 (275.3)** | 4551.3 | 4940.7 | 0.0008 |
|  | C24 | **888.5 (136.6)** | 985.0 | 791.9 | **586.8 (31.1)** | 608.8 | 564.8 |  | **634.9 (89.8)** | 698.3 | 571.4 |  |
|  | C24:1 | **14337.9 (2312.9)** | 15973.3 | 12702.5 | ***9561.7 (486.4)** | 9905.7 | 9217.7 | <0.0001 | ***10397.4 (1241.4)** | 11275.2 | 9519.5 | <0.0001 |
|  | C26 | **4.5 (2.5)** | 6.3 | 2.8 | **3.8 (0.5)** | 3.4 | 4.1 |  | **2.6 (0.1)** | 2.5 | 2.7 |  |
|  | C26:1 | **51.5 (7.3)** | 56.7 | 46.3 | **43.1 (4.1)** | 40.2 | 46.0 |  | **42.6 (16.6)** | 54.3 | 30.9 |  |
|  | Lyso | **0.1 (0.1)** | 0.2^a^ | 0.1^a^ | **0.2 (0.1)** | 0.2 | 0.1^a^ |  | **0.2 (0.1)** | 0.1^a^ | 0.2 |  |
|  |  |  |  |  |  |  |  |  |  |  |  |  |
| lymph | C14 | **81.9 (4.0)** | 79.1 | 84.8 | **101.4 (30.4)** | 122.8 | 79.9^a^ |  | **65.9 (16.0)** | 77.2 | 54.6^a^ |  |
|  | C16 | **2044.5 (16.2)** | 2056.0 | 2033.0 | ***1389.8 (376.4)** | 1123.6 | 1656.0 | 0.0112 | **1836.0 (5.7)** | 1840.0 | 1831.9 |  |
|  | C18 | **480.6 (363.8)** | 737.9 | 223.4 | **186.0 (15.0)** | 175.4 | 196.5 |  | **247.1 (13.6)** | 256.8 | 237.5 |  |
|  | C18:1 | **1855.0 (131.5)** | 1948.0 | 1762.0 | **1516.9 (228.7)** | 1355.2 | 1678.6 |  | **1918.5 (37.8)** | 1891.8 | 1945.3 |  |
|  | C20 | **992.8 (141.8)** | 1093.0 | 892.5 | **931.3 (137.2)** | 1028.3 | 834.3 |  | **710.8 (51.5)** | 747.2 | 674.4 |  |
|  | C20:1 | **2469.8 (404.2)** | 2184.0 | 2755.6 | **2143.2 (27.6)** | 2162.8 | 2123.7 |  | **2035.1 (54.4)** | 2073.6 | 1996.6 |  |
|  | C22 | **530.1 (125.3)** | 618.6 | 441.5^a^ | **414.7 (58.2)** | 455.8^a^ | 373.5^a^ |  | **458.3 (101.4)** | 530.0 | 386.6^a^ |  |
|  | C22:1 | **4788.4 (520.9)** | 5156.8 | 4420.1 | ***3500.3 (179.7)** | 3627.4 | 3373.2 | <0.0001 | **6095.6 (2978.9)** | 8202.1 | 3989.2 |  |
|  | C24 | **250.1 (89.0)** | 313.1 | 187.2 | **125.5 (16.2)** | 137.0 | 114.1 |  | **523.9 (387.8)** | 798.1 | 249.7 |  |
|  | C24:1 | **5281.3 (586.6)** | 5696.2 | 4866.5 | ***3570.7 (370.8)** | 3308.5 | 3832.8 | <0.0001 | ***11232.9 (8591.5)** | 17308.0 | 5157.8 | 0.0033 |
|  | C26 | **19.2 (1.8)** | 20.5 | 17.9 | **21.5 (6.9)** | 26.4 | 16.7 |  | **18.7 (0.7)** | 18.2 | 19.2 |  |
|  | C26:1 | **17.1 (6.3)** | 21.5 | 12.7 | **20.9 (11.7)** | 29.2 | 12.7 |  | **26.0 (15.4)** | 36.9 | 15.1 |  |
|  | Lyso | **––** | 1.2^b^ | c | **––** | 1.1^b^ | 2.1^b^ |  | **4.4 (2.3)** | 2.7^a^ | 6.0 |  |

| **Supplemental Table 10: Sphingoid base (SB) species blood:lymph ratios** | | | | | | | | | | | |
| --- | --- | --- | --- | --- | --- | --- | --- | --- | --- | --- | --- |
| Blood:lymph ratios for individual samples (“A” and “B”) are obtained by dividing the nM blood concentration by the respective lymph nM concentration. The individual blood:lymph ratios are averaged to yield the mean for each species. WT = wild-type, KO = *Apom^-/-^*, Tg = *APOM* transgenic mice.  *Mean is statistically different from WT mean. a: At least one value used to compute the ratio is from a sample whose signal was one to two times that of the matrix blank. b: At least one value used to compute the ratio is from a sample whose signal was below that of the matrix blank. The ratio is excluded from further analysis.  c: At least one value used to compute the ratio is from a sample whose signal was below the detection limit and thus the ratio is undefined. | | | | | | | | | | | |
|  | **WT** | | | **KO** | | | | **Tg** | | | |
| **species** | **mean (SD)** | A | B | **mean (SD)** | A | B | *p*-value | **mean (SD)** | A | B | *p*-value |
| S1P | **10.354 (2.378)** | 12.036 | 8.673 | ***2.355 (0.337)** | 2.594 | 2.117 | 0.0006 | **10.049 (3.889)** | 12.799 | 7.299 |  |
| dhS1P | **––** | 6.602^b^ | c | **––** | 7.468^b^ | 3.837^b^ |  | **––** | 37.263^b^ | c |  |
| Sph | **0.536 (0.145)** | 0.433 | 0.639^a^ | **0.878 (0.430)** | 1.182^a^ | 0.574 |  | **0.332 (0.315)** | 0.554^a^ | 0.109 |  |
| dhSph | **0.259 (0.000)** | 0.259^a^ | 0.259^a^ | **0.370 (0.067)** | 0.418^a^ | 0.323^a^ |  | **0.125** | 0.528^b^ | 0.125^a^ |  |

| **Supplemental Table 11: Ceramide (Cer) species blood:lymph ratios** | | | | | | | | | | | |
| --- | --- | --- | --- | --- | --- | --- | --- | --- | --- | --- | --- |
| Blood:lymph ratios for individual samples (“A” and “B”) are obtained by dividing the nM blood concentration by the respective lymph nM concentration. The individual blood:lymph ratios are averaged to yield the mean for each species. WT = wild-type, KO = *Apom^-/-^*, Tg = *APOM* transgenic mice.  *Mean is statistically different from WT mean. a: At least one value used to compute the ratio is from a sample whose signal was one to two times that of the matrix blank. b: At least one value used to compute the ratio is from a sample whose signal was below that of the matrix blank. The ratio is excluded from further analysis.  c: At least one value used to compute the ratio is from a sample whose signal was below the detection limit and thus the ratio is undefined. | | | | | | | | | | | |
|  | **WT** | | | **KO** | | | | **Tg** | | | |
| **species (nM)** | **mean (SD)** | A | B | **Mean (SD)** | A | B | *p*-value | **mean (SD)** | A | B | *p*-value |
| C14 | **0.030 (0.014)** | 0.040 | 0.019^a^ | **0.017 (0.012)** | 0.025^a^ | 0.008^a^ |  | **0.038** | 0.038 | 0.007^b^ |  |
| C16 | **0.022 (0.012)** | 0.031 | 0.014 | **0.033 (0.005)** | 0.037 | 0.030 |  | **0.034 (0.007)** | 0.029 | 0.040 |  |
| C18 | **0.075 (0.001)** | 0.075^a^ | 0.076 | **0.032** | 0.110^b^ | 0.032 |  | **0.179 (0.092)** | 0.114^a^ | 0.244^a^ |  |
| C18:1 | **0.040 (0.001)** | 0.040 | 0.039 | **0.072 (0.085)** | 0.132 | 0.012 |  | **0.117 (0.038)** | 0.144 | 0.090 |  |
| C20 | **0.148 (0.006)** | 0.152^a^ | 0.144^a^ | **0.086 (0.090)** | 0.150^a^ | 0.022 |  | **––** | 0.384^b^ | 0.571^b^ |  |
| C20:1 | **0.058 (0.054)** | 0.096 | 0.020 | **0.048 (0.039)** | 0.076 | 0.021 |  | **0.044 (0.042)** | 0.014 | 0.074 |  |
| C20:4 | **––** | c | c | **––** | c | c |  | **––** | c | c |  |
| C22 | **0.278 (0.143)** | 0.379 | 0.177 | **0.366 (0.248)** | 0.541 | 0.191 |  | **0.406 (0.154)** | 0.297 | 0.514 |  |
| C22:1 | **0.086 (0.022)** | 0.101 | 0.070 | **0.099 (0.070)** | 0.148 | 0.049 |  | **0.102 (0.040)** | 0.074 | 0.131 |  |
| C24 | **0.083 (0.012)** | 0.091 | 0.074 | **0.147 (0.096)** | 0.215 | 0.079 |  | **0.154 (0.063)** | 0.109 | 0.199 |  |
| C24:1 | **1.045 (0.197)** | 1.184 | 0.906 | **1.677 (0.953)** | 2.351 | 1.003 |  | ***1.657 (0.002)** | 1.655 | 1.659 | 0.0479 |
| C26 | **––** | 0.386^b^ | 0.094^b^ | **0.046** | 0.135^b^ | 0.046^a^ |  | **––** | 0.109^b^ | 0.527^b^ |  |
| C26:1 | **0.388 (0.145)** | 0.285^a^ | 0.490^a^ | **0.055 (0.041)** | 0.084 | 0.026 |  | **0.395 (0.225)** | 0.554^a^ | 0.236 |  |

| **Supplemental Table 12: Dihydroceramide (dhCer) species blood:lymph ratios** | | | | | | | | | | | |
| --- | --- | --- | --- | --- | --- | --- | --- | --- | --- | --- | --- |
| Blood:lymph ratios for individual samples (“A” and “B”) are obtained by dividing the nM blood concentration by the respective lymph nM concentration. The individual blood:lymph ratios are averaged to yield the mean for each species. WT = wild-type, KO = *Apom^-/-^*, Tg = *APOM* transgenic mice.  *Mean is statistically different from WT mean. a: At least one value used to compute the ratio is from a sample whose signal was one to two times that of the matrix blank. b: A t least one value used to compute the ratio is from a sample whose signal was below that of the matrix blank. The ratio is excluded from further analysis.  c: At least one value used to compute the ratio is from a sample whose signal was below the detection limit and thus the ratio is undefined. | | | | | | | | | | | |
|  | **WT** | | | **KO** | | | | **Tg** | | | |
| **species (nM)** | **mean (SD)** | A | B | **mean (SD)** | A | B | *p*-value | **mean (SD)** | A | B | *p*-value |
| C12 | **0.081** | 0.051^b^ | 0.081^a^ | **0.002** | 0.002^a^ | 0.017^b^ |  | **––** | 0.476^b^ | 0.666^b^ |  |
| C14 | **0.034 (0.013)** | 0.044 | 0.025 | **0.064** | 0.675^b^ | 0.064 |  | **0.170 (0.131)** | 0.263 | 0.077 |  |
| C16 | **0.023 (0.012)** | 0.031 | 0.014 | **0.044 (0.026)** | 0.062 | 0.026 |  | **0.025 (0.004)** | 0.028 | 0.022 |  |
| C18 | **0.039 (0.006)** | 0.043 | 0.035^a^ | **0.037** | 0.087^b^ | 0.037 |  | **0.113** | 0.113^a^ | 0.183^b^ |  |
| C18:1 | **0.073 (0.071)** | 0.123^a^ | 0.022^a^ | **0.088 (0.053)** | 0.051^a^ | 0.125 |  | **––** | 1.106^b^ | 0.434^b^ |  |
| C20 | **0.030 (0.022)** | 0.045 | 0.014 | **0.039 (0.041)** | 0.069 | 0.010 |  | **0.111 (0.004)** | 0.109 | 0.114 |  |
| C20:1 | **0.021 (0.008)** | 0.027 | 0.015 | **0.037 (0.010)** | 0.030 | 0.045 |  | **0.106 (0.054)** | 0.068 | 0.144 |  |
| C22 | **0.026 (0.015)** | 0.036 | 0.015 | **0.048 (0.012)** | 0.057 | 0.040 |  | **0.062 (0.015)** | 0.052 | 0.073 |  |
| C22:1 | **0.367 (0.197)** | 0.507 | 0.228 | **0.483 (0.334)** | 0.719 | 0.247 |  | **0.532 (0.206)** | 0.386 | 0.677 |  |
| C24 | **0.041 (0.017)** | 0.053 | 0.029 | **0.074 (0.025)** | 0.091 | 0.056 |  | **0.088 (0.024)** | 0.072 | 0.105 |  |
| C24:1 | **0.857 (0.271)** | 1.049 | 0.665 | **1.148 (0.877)** | 1.768 | 0.528 |  | ***1.296 (0.293)** | 1.089 | 1.502 | 0.0021 |
| C26 | **0.085** | 0.085^a^ | 0.031^b^ | **0.031** | 0.068^b^ | 0.031 |  | **0.109** | 0.125^b^ | 0.109^a^ |  |
| C26:1 | **––** | 0.321^b^ | 0.215^b^ | **0.029** | 0.253^b^ | 0.029^a^ |  | **––** | 0.448^b^ | 0.301^b^ |  |

| **Supplemental Table 13: Alpha-hydroxyceramide (αOHCer) species blood:lymph ratios** | | | | | | | | | | | |
| --- | --- | --- | --- | --- | --- | --- | --- | --- | --- | --- | --- |
| Blood:lymph ratios for individual samples (“A” and “B”) are obtained by dividing the nM blood concentration by the respective lymph nM concentration. The individual blood:lymph ratios are averaged to yield the mean for each species. WT = wild-type, KO = *Apom^-/-^*, Tg = *APOM* transgenic mice.  *Mean is statistically different from WT mean. a: At least one value used to compute the ratio is from a sample whose signal was one to two times that of the matrix blank. b: At least one value used to compute the ratio is from a sample whose signal was below that of the matrix blank. The ratio is excluded from further analysis.  c: At least one value used to compute the ratio is from a sample whose signal was below the detection limit and thus the ratio is undefined. | | | | | | | | | | | |
|  | **WT** | | | **KO** | | | | **Tg** | | | |
| **species (nM)** | **mean (SD)** | A | B | **mean (SD)** | A | B | *p*-value | **mean (SD)** | A | B | *p*-value |
| C14 | **0.021 (0.004)** | 0.018 | 0.024 | **0.027 (0.001)** | 0.026 | 0.028 |  | **0.038 (0.004)** | 0.041 | 0.034 |  |
| C16 | **0.013 (0.001)** | 0.013 | 0.014 | **0.011 (0.002)** | 0.010 | 0.012 |  | **0.017 (0.004)** | 0.014 | 0.020 |  |
| C18 | **0.026 (0.005)** | 0.022 | 0.029 | ***0.064 (0.006)** | 0.068 | 0.060 | 0.0118 | ***0.087 (0.022)** | 0.072 | 0.103 | 0.0006 |
| C18:1 | **0.005** | 0.002^b^ | 0.005 | **0.013 (0.002)** | 0.012 | 0.015 |  | **0.011** | 0.003^b^ | 0.011 |  |
| C20 | **0.001 (0.001)** | 0.002^a^ | 0.001^a^ | **0.003** | 0.003 | 0.002^b^ |  | **0.003 (0.000)** | 0.003^a^ | 0.003 |  |
| C20:1 | **––** | 0.002^b^ | 0.001^b^ | **0.007 (0.002)** | 0.005^a^ | 0.008^a^ |  | **––** | 0.003^b^ | 0.003^b^ |  |
| C22 | **0.014 (0.000)** | 0.014 | 0.015 | ***0.044 (0.008)** | 0.038 | 0.049 | 0.0473 | **0.025 (0.009)** | 0.019 | 0.032 |  |
| C22:1 | **0.028 (0.000)** | 0.028 | 0.028 | **0.032 (0.000)** | 0.032 | 0.033 |  | ***0.066 (0.021)** | 0.051 | 0.081 | 0.0214 |
| C24 | **0.004 (0.002)** | 0.005 | 0.003^a^ | **0.006 (0.003)** | 0.008^a^ | 0.005^a^ |  | **0.010 (0.003)** | 0.013 | 0.008 |  |
| C24:1 | **0.156 (0.018)** | 0.169 | 0.143 | ***0.080 (0.039)** | 0.108 | 0.052 | <0.0001 | ***0.192 (0.026)** | 0.174 | 0.210^a^ | 0.0278 |
| C26 | **––** | 0.0003^b^ | c | **––** | 0.004^b^ | 0.001^b^ |  | **0.005** | 0.001^b^ | 0.005^a^ |  |
| C26:1 | **0.020 (0.001)** | 0.019 | 0.020 | ***0.068 (0.040)** | 0.040 | 0.096 | 0.0024 | ***0.082 (0.044)** | 0.051 | 0.113 | 0.0006 |

| **Supplemental Table 14: Ceramide 1-phosphate (C1P) species blood:lymph ratios** | | | | | | | | | | | |
| --- | --- | --- | --- | --- | --- | --- | --- | --- | --- | --- | --- |
| Blood:lymph ratios for individual samples (“A” and “B”) are obtained by dividing the nM blood concentration by the respective lymph nM concentration. The individual blood:lymph ratios are averaged to yield the mean for each species. WT = wild-type, KO = *Apom^-/-^*, Tg = *APOM* transgenic mice.  a: At least one value used to compute the ratio is from a sample whose signal was one to two times that of the matrix blank. b: At least one value used to compute the ratio is from a sample whose signal was below that of the matrix blank. The ratio is excluded from further analysis. c: At least one value used to compute the ratio is from a sample whose signal was below the detection limit and thus the ratio is undefined. | | | | | | | | | | | |
|  | **WT** | | | **KO** | | | | **Tg** | | | |
| **species (nM)** | **mean (SD)** | A | B | **Mean (SD)** | A | B | *p*-value | **mean (SD)** | A | B | *p*-value |
| C14 | **0.025 (0.006)** | 0.029 | 0.020 | **0.023 (0.021)** | 0.038 | 0.008 |  | **0.039 (0.016)** | 0.028 | 0.051 |  |
| C16 | **0.131 (0.019)** | 0.144 | 0.117 | **0.149 (0.062)** | 0.193 | 0.105 |  | **0.147 (0.077)** | 0.093 | 0.202 |  |
| C18 | **0.249 (0.067)** | 0.296 | 0.202 | **0.304 (0.309)** | 0.522 | 0.086 |  | **0.281 (0.131)** | 0.188 | 0.374 |  |
| C18:1 | **0.513 (0.367)** | 0.772^a^ | 0.254 | **0.380 (0.394)** | 0.659^a^ | 0.101 |  | **0.602 (0.439)** | 0.291 | 0.913 |  |
| C20 | **0.225** | 0.225^a^ | 0.160^b^ | **0.158 (0.190)** | 0.293^a^ | 0.024 |  | **0.122 (0.003)** | 0.120^a^ | 0.125^a^ |  |
| C20:1 | **––** | c | c | **––** | c | c |  | **––** | c | c |  |
| C22 | **0.030 (0.023)** | 0.013 | 0.046 | **0.031 (0.029)** | 0.051 | 0.011 |  | **0.034 (0.025)** | 0.016 | 0.052^a^ |  |
| C22:1 | **––** | c | c | **0.355 (0.502)** | 0.710^a^ | c |  | **0.055 (0.028)** | 0.035 | 0.075 |  |
| C24 | **0.039 (0.001)** | 0.040 | 0.038 | **0.017 (0.017)** | 0.029 | 0.005 |  | **0.030 (0.013)** | 0.020 | 0.039 |  |
| C24:1 | **0.044** | 0.044^a^ | 0.183^b^ | **0.078** | 0.039^b^ | 0.078 |  | **0.022** | 0.022^a^ | 0.078^b^ |  |
| C26 | **0.038 (0.003)** | 0.036 | 0.040 | **0.024 (0.011)** | 0.032 | 0.017 |  | **0.054 (0.008)** | 0.049 | 0.060 |  |
| C26:1 | **0.026** | 0.026^a^ | 0.030^b^ | **0.014** | 0.023^b^ | 0.014^a^ |  | **0.051 (0.032)** | 0.028^a^ | 0.074^a^ |  |

| **Supplemental Table 15: Diacylglycerol (DAG) species blood:lymph ratios** | | | | | | | | | | | |
| --- | --- | --- | --- | --- | --- | --- | --- | --- | --- | --- | --- |
| Blood:lymph ratios for individual samples (“A” and “B”) are obtained by dividing the nM blood concentration by the respective lymph nM concentration. The individual blood:lymph ratios are averaged to yield the mean for each species. WT = wild-type, KO = *Apom^-/-^*, Tg = *APOM* transgenic mice.  *Mean is statistically different from WT mean. a: At least one value used to compute the ratio is from a sample whose signal was one to two times that of the matrix blank. b: At least one value used to compute the ratio is from a sample whose signal was below that of the matrix blank. The ratio is excluded from further analysis.  c: At least one value used to compute the ratio is from a sample whose signal was below the detection limit and thus the ratio is undefined. | | | | | | | | | | | |
|  | **WT** | | | **KO** | | | | **Tg** | | | |
| **species (nM)** | **mean (SD)** | A | B | **mean (SD)** | A | B | *p*-value | **mean (SD)** | A | B | *p*-value |
| di-C14 | **0.023 (0.012)** | 0.014 | 0.031 | **0.053 (0.054)** | 0.015 | 0.091 |  | **0.044 (0.012)** | 0.053 | 0.035 |  |
| C14:0/16:0 | **0.011 (0.005)** | 0.008 | 0.015 | **0.025 (0.021)** | 0.011 | 0.040 |  | **0.035 (0.006)** | 0.039 | 0.031 |  |
| C14:0/18:0 | **0.011 (0.008)** | 0.006 | 0.017 | **0.017 (0.014)** | 0.007 | 0.027 |  | **0.028 (0.003)** | 0.030 | 0.026 |  |
| C14:0/18:1 | **0.007 (0.000)** | 0.007 | 0.007 | **0.013 (0.001)** | 0.014 | 0.013 |  | **0.016 (0.014)** | 0.006 | 0.026 |  |
| di-C16:0 | **0.008 (0.002)** | 0.006 | 0.010 | **0.016 (0.011)** | 0.009 | 0.024 |  | **0.021 (0.002)** | 0.020 | 0.023 |  |
| C16:0/18:0 | **0.013 (0.006)** | 0.009 | 0.017 | **0.024 (0.020)** | 0.009 | 0.038 |  | **0.023 (0.002)** | 0.022 | 0.025 |  |
| C16:0/18:1 | **0.010 (0.001)** | 0.011 | 0.008 | **0.020 (0.011)** | 0.028 | 0.012 |  | **0.032 (0.025)** | 0.014 | 0.050 |  |
| C16:0/24:1 | **0.055 (0.045)** | 0.087 | 0.023 | **0.033 (0.018)** | 0.046 | 0.021 |  | **0.046 (0.061)** | 0.003^a^ | 0.089 |  |
| di-C16:1 | **0.024 (0.003)** | 0.022 | 0.026 | **0.043 (0.012)** | 0.035 | 0.052 |  | **0.040 (0.002)** | 0.041 | 0.039 |  |
| C16:1/18:0 | **0.010 (0.002)** | 0.009 | 0.012 | **0.011 (0.001)** | 0.010 | 0.012 |  | **0.022 (0.016)** | 0.011 | 0.033 |  |
| C16:1/18:1 | **0.028 (0.009)** | 0.034 | 0.022 | **0.040 (0.015)** | 0.051 | 0.029 |  | **0.053 (0.032)** | 0.030 | 0.076 |  |
| C16:1/20:0 | **0.005** | 0.0005^b^ | 0.005 | **0.010** | 0.004^b^ | 0.010 |  | **0.005 (0.002)** | 0.003 | 0.006 |  |
| C16:1/24:1 | **––** | c | 0.019^b^ | **––** | c | c |  | **––** | 0.229^b^ | c |  |
| di-C18:0 | **0.018 (0.011)** | 0.010 | 0.026 | **0.022 (0.017)** | 0.010 | 0.035 |  | **0.029 (0.003)** | 0.030 | 0.027 |  |
| C18:0/18:1 | **0.008 (0.001)** | 0.007 | 0.008 | **0.017 (0.000)** | 0.017 | 0.017 |  | **0.022 (0.019)** | 0.009 | 0.036 |  |
| C18:0/18:2 | **0.003 (0.001)** | 0.004 | 0.003 | **0.009 (0.002)** | 0.011 | 0.007 |  | **0.007 (0.003)** | 0.004 | 0.009 |  |
| C18:0/20:4 | **0.024 (0.005)** | 0.021 | 0.028 | ***0.090 (0.045)** | 0.122 | 0.058 | 0.0011 | **0.032 (0.005)** | 0.029 | 0.035 |  |
| di-C18:1 | **0.018 (0.000)** | 0.019 | 0.018 | **0.047 (0.002)** | 0.049 | 0.046 |  | **0.035 (0.022)** | 0.020 | 0.051 |  |
| C18:1/24:0 | **0.014 (0.007)** | 0.009 | 0.019 | **0.019 (0.010)** | 0.012 | 0.026 |  | **0.067** | 0.276^b^ | 0.067 |  |
| C18:1/24:1 | **0.009 (0.008)** | 0.014 | 0.004 | ***0.091 (0.051)** | 0.055 | 0.128 | <0.0001 | ***0.222 (0.109)** | 0.145 | 0.299 | <0.0001 |

| **Supplemental Table 16: Sphingomyelin (SM) species blood:lymph ratios** | | | | | | | | | | | |
| --- | --- | --- | --- | --- | --- | --- | --- | --- | --- | --- | --- |
| Blood:lymph ratios for individual samples (“A” and “B”) are obtained by dividing the nM blood concentration by the respective lymph nM concentration. The individual blood:lymph ratios are averaged to yield the mean for each species. WT = wild-type, KO = *Apom^-/-^*, Tg = *APOM* transgenic mice.  *Mean is statistically different from WT mean. a: At least one value used to compute the ratio is from a sample whose signal was one to two times that of the matrix blank. b: At least one value used to compute the ratio is from a sample whose signal was below that of the matrix blank. The ratio is excluded from further analysis.  c: At least one value used to compute the ratio is from a sample whose signal was below the detection limit and thus the ratio is undefined. | | | | | | | | | | | |
|  | **WT** | | | **KO** | | | | **Tg** | | | |
| **species (nM)** | **mean (SD)** | A | B | **Mean (SD)** | A | B | *p*-value | **mean (SD)** | A | B | *p*-value |
| C14 | **3.611 (1.528)** | 4.691 | 2.531 | **2.350 (0.411)** | 2.059 | 2.640 |  | **15.152 (16.894)** | 27.099 | 3.206 |  |
| C16 | **4.420 (1.235)** | 5.294 | 3.547 | **7.081 (2.285)** | 8.696 | 5.465 |  | **4.890 (0.302)** | 5.104 | 4.676 |  |
| C18 | **2.692 (0.741)** | 3.216 | 2.168 | **1.907 (1.235)** | 1.033 | 2.780 |  | **1.609 (1.689)** | 2.804 | 0.415 |  |
| C18:1 | **64.321 (82.235)** | 122.469 | 6.172 | **7.191 (1.507)** | 8.256 | 6.125 |  | **42.644 (1.089)** | 41.873 | 43.414 |  |
| C20 | **1.094 (0.124)** | 1.006 | 1.182 | **9.309 (10.615)** | 1.803 | 16.815 |  | **2.285 (1.194)** | 1.441 | 3.129 |  |
| C20:1 | **––** | c | 345.000^b^ | **––** | c | c |  | **27.926 (3.452)** | 30.367^b^ | 25.485^b^ |  |
| C22 | **76.002 (33.996)** | 100.040 | 51.963 | **98.753 (110.077)** | 20.917 | 176.589 |  | **12.734 (10.695)** | 5.172 | 20.297 |  |
| C22:1 | **176.826 (14.857)** | 166.321 | 187.331 | **140.956 (139.872)** | 42.052 | 239.861 |  | ***10.472 (2.501)** | 12.241 | 8.703 | 0.0041 |
| C24 | **49.109 (27.896)** | 68.835 | 29.383 | **86.376 (0.003)** | 86.378 | 86.373 |  | **17.664 (1.292)** | 18.578 | 16.751 |  |
| C24:1 | **455.680 (516.727)** | 90.299 | 821.061 | **190.757 (189.759)** | 56.577 | 324.937 |  | **66.599 (34.037)** | 42.531 | 90.666 |  |
| C26 | **––** | c | 0.643^b^ | **––** | 0.333^b^ | c |  | **0.152 (0.104)** | 0.078 | 0.226^a^ |  |
| C26:1 | **––** | c | c | **––** | 0.267^b^ | c |  | **0.186 (0.263)** | c | 0.373 |  |
| Lyso | **––** | 0.302^b^ | c | **––** | 0.021^b^ | c |  | **––** | c | c |  |

| **Supplemental Table 17: Dihydrosphingomyelin (dhSM) species blood:lymph ratios** | | | | | | | | | | | |
| --- | --- | --- | --- | --- | --- | --- | --- | --- | --- | --- | --- |
| Blood:lymph ratios for individual samples (“A” and “B”) are obtained by dividing the nM blood concentration by the respective lymph nM concentration. The individual blood:lymph ratios are averaged to yield the mean for each species. WT = wild-type, KO = *Apom^-/-^*, Tg = *APOM* transgenic mice.  *Mean is statistically different from WT mean. a: At least one value used to compute the ratio is from a sample whose signal was one to two times that of the matrix blank. b: At least one value used to compute the ratio is from a sample whose signal was below that of the matrix blank. The ratio is excluded from further analysis.  c: At least one value used to compute the ratio is from a sample whose signal was below the detection limit and thus the ratio is undefined. | | | | | | | | | | | |
|  | **WT** | | | **KO** | | | | **Tg** | | | |
| **species (nM)** | **mean (SD)** | A | B | **mean (SD)** | A | B | *p*-value | **mean (SD)** | A | B | *p*-value |
| C14 | **0.177 (0.039)** | 0.205 | 0.150 | **0.209 (0.050)** | 0.173 | 0.244^a^ |  | **0.294 (0.027)** | 0.275 | 0.313^a^ |  |
| C16 | **0.595 (0.104)** | 0.669 | 0.521 | **0.912 (0.290)** | 1.117 | 0.707 |  | **0.666 (0.064)** | 0.711 | 0.621 |  |
| C18 | **0.447 (0.311)** | 0.227 | 0.667 | **0.955 (0.007)** | 0.950 | 0.960 |  | **0.626 (0.072)** | 0.575 | 0.677 |  |
| C18:1 | **0.833 (0.049)** | 0.868 | 0.798 | **1.229 (0.049)** | 1.264 | 1.194 |  | **0.913 (0.091)** | 0.848 | 0.977 |  |
| C20 | **0.106 (0.010)** | 0.099 | 0.113 | **0.104 (0.018)** | 0.091 | 0.117 |  | **0.114 (0.011)** | 0.106 | 0.121 |  |
| C20:1 | **0.227 (0.049)** | 0.262 | 0.192 | **0.256 (0.018)** | 0.243 | 0.268 |  | **0.214 (0.014)** | 0.204 | 0.224 |  |
| C22 | **1.116 (0.003)** | 1.114 | 1.119^a^ | **1.105 (0.046)** | 1.073^a^ | 1.137^a^ |  | **0.899 (0.201)** | 0.757 | 1.041^a^ |  |
| C22:1 | **1.478 (0.216)** | 1.631 | 1.325 | **1.621 (0.166)** | 1.739 | 1.504 |  | **0.897 (0.483)** | 0.555 | 1.239 |  |
| C24 | **3.688 (0.767)** | 3.146 | 4.230 | ***4.697 (0.358)** | 4.444 | 4.950 | 0.0246 | ***1.582 (0.999)** | 0.875 | 2.288 | <0.0001 |
| C24:1 | **2.707 (0.137)** | 2.804 | 2.610 | **2.699 (0.417)** | 2.994 | 2.405 |  | ***1.249 (0.844)** | 0.651 | 1.846 | 0.0006 |
| C26 | **0.232 (0.107)** | 0.307 | 0.156 | **0.187 (0.083)** | 0.129 | 0.246 |  | **0.139 (0.002)** | 0.137 | 0.141 |  |
| C26:1 | **3.141 (0.713)** | 2.637 | 3.646 | **2.499 (1.588)** | 1.377 | 3.622 |  | ***1.759 (0.406)** | 1.472 | 2.046 | 0.0010 |
| Lyso | **––** | 0.167^b^ | c | **––** | 0.182^b^ | 0.048^b^ |  | **0.035 (0.003)** | 0.037^a^ | 0.033 |  |
